# Taxane chemotherapy induces stromal injury that leads to breast cancer dormancy escape

**DOI:** 10.1371/journal.pbio.3002275

**Published:** 2023-09-12

**Authors:** Ramya Ganesan, Swati S. Bhasin, Mojtaba Bakhtiary, Upaasana Krishnan, Nagarjuna R. Cheemarla, Beena E. Thomas, Manoj K. Bhasin, Vikas P. Sukhatme

**Affiliations:** 1 Department of Medicine—Renal Division, Emory University School of Medicine, Atlanta, Georgia, United States of America; 2 Department of Pediatrics—Hematology Division, Emory University School of Medicine, Atlanta, Georgia, United States of America; 3 Aflac Cancer and Blood Disorders Center, Children’s Healthcare of Atlanta, Atlanta, Georgia, United States of America; 4 Department of Biomedical Engineering, Georgia Institute of Technology, Atlanta, Georgia, United States of America; 5 Department of Laboratory Medicine, Yale School of Medicine, New Haven, Connecticut, United States of America; 6 Department of Immunobiology, Yale School of Medicine, New Haven, Connecticut, United States of America; 7 Department of Biomedical Informatics, Emory University, Atlanta, Georgia, United States of America; 8 Department of Hematology and Medical Oncology, Emory University School of Medicine, Atlanta, Georgia, United States of America; 9 Winship Cancer Institute, Emory University, Atlanta, Georgia, United States of America; UC Los Angeles: University of California Los Angeles, UNITED STATES

## Abstract

A major cause of cancer recurrence following chemotherapy is cancer dormancy escape. Taxane-based chemotherapy is standard of care in breast cancer treatment aimed at killing proliferating cancer cells. Here, we demonstrate that docetaxel injures stromal cells, which release protumor cytokines, IL-6 and granulocyte colony stimulating factor (G-CSF), that in turn invoke dormant cancer outgrowth both in vitro and in vivo. Single-cell transcriptomics shows a reprogramming of awakened cancer cells including several survival cues such as stemness, chemoresistance in a tumor stromal organoid (TSO) model, as well as an altered tumor microenvironment (TME) with augmented protumor immune signaling in a syngeneic mouse breast cancer model. IL-6 plays a role in cancer cell proliferation, whereas G-CSF mediates tumor immunosuppression. Pathways and differential expression analyses confirmed MEK as the key regulatory molecule in cancer cell outgrowth and survival. Antibody targeting of protumor cytokines (IL-6, G-CSF) or inhibition of cytokine signaling via MEK/ERK pathway using selumetinib prior to docetaxel treatment prevented cancer dormancy outgrowth suggesting a novel therapeutic strategy to prevent cancer recurrence.

## Abstract 2

Cancer dormancy is a major hindrance to achieving complete clinical response in patients. This study uses a tumor stromal organoid and murine breast cancer model to reveal that chemotherapy injures stromal cells, but not dormant cancer cells directly; this stromal injury in turn invokes proinflammatory cytokine signaling and can awaken dormant cancer cells.

## Introduction

Cancer that is locoregional is typically treated with surgery and with neoadjuvant or adjuvant chemotherapy, radiation therapy or immunotherapy to prevent local and most importantly systemic recurrences. One mechanism for the latter is the awakening of cancer cells that are in a dormant state, which may not be eradicated by neoadjuvant or adjuvant treatments. Dormancy can be cellular (single dormant cells) or occur as a cluster wherein proliferation is balanced by cell death. Earlier studies have shown that surgery itself promotes early dormancy escape and accelerates relapse [1,2]. Recent studies have shown that inhibiting the surgery-mediated injury response by administering preoperative NSAIDs can eradicate micrometastases that exist at the time of surgery [3,4]. More recent studies have shown the role of chronic inflammation, injury response, fibrosis and autophagy inhibition in awakening, or elimination of dormant cancer cells [3,5–8].

Chemotherapy is given in neoadjuvant and adjuvant settings, as well as standalone therapy to reduce tumor burden. However, in addition to the known toxicities of such drugs, there is an increased appreciation of other deleterious effects of such treatments. Docetaxel (T), a second-generation taxane, is given to breast cancer patients as a single agent (doses ranging from 60 to 100 mg/m^2^) or in combination with other chemotherapeutics such as doxorubicin (A) and cytoxan (C), in cycles. Some preclinical studies have shown the effects of chemotherapy in inducing dormancy [9,10], while others have focused on stress, senescence-associated secretory phenotype and chemotherapy-induced cell debris in cancer dormancy awakening, tumor growth, and metastasis [11–14]. To date the mechanistic basis for the effects of chemotherapy on cancer dormancy in vivo has not been elucidated.

Using a model of breast cancer dormancy, we show for the first time that taxane-based chemotherapy induces proinflammatory cytokine release by injured stromal cells and awakens dormant cancer cells potentiating aggressive immune evasion and cancer outgrowth. We find that inhibiting IL-6 and/or granulocyte colony stimulating factor (G-CSF)-mediated MEK signaling cascade prevents chemotherapy-induced cancer dormancy escape.

## Materials and methods

### Ethics statement

The mice used in this study were housed at the animal facility of Emory University. Strict adherence to the Institutional Animal Care and Use Committee (IACUC) guidelines ensured the highest standards of animal welfare. The Emory IACUC granted approval for the animal experiments performed in this research under the protocol number PROTO201800020.

### Cell lines

D2.0R-luc-mcherry and D2.0R-FUCCI cells were obtained from Dr. Mikala Egeblad’s lab in Cold Spring Harbor Laboratory [5]. 2H11 and murine embryonic fibroblast (MEF) cells are murine endothelial and fibroblast cells lines, respectively, purchased from ATCC. All cell lines were cultured in DMEM supplemented with 10% FBS, 100 U/mL penicillin, 100 μg/mL streptomycin, 4.5 g/L glucose, 4 mM L-Glutamine, 1 mM sodium pyruvate, and 1.5 g/L sodium bicarbonate. All cell cultures were passaged at 80% confluency and tested negative for murine pathogens, including mycoplasma. All cells were maintained in a humidified 37°C CO_2_ incubator.

### In vitro tumor stromal organoid dormancy model

D2.0R-luc-mCherry were cocultured in reduced growth factor BME with murine endothelial cells (2H11) and/or MEFs in DMEM supplemented with 2% FBS, 100 U/mL penicillin, 100 μg/mL streptomycin, 1 g/L glucose, 4 mM L-Glutamine, 1 mM sodium pyruvate, and 1,500 mg/L sodium bicarbonate. The tumor stromal organoid (TSO) was incubated for 3 to 4 days in RGF BME-coated plates to establish cancer dormancy, followed by treatment with vehicle, docetaxel, selumetinib, anti-IL-6 Ab, anti-G-CSF Ab, or combinations. At the end of treatment, cells were harvested for flow cytometry staining.

### EdU incorporation

TSO culture was established as described above in RGF BME-coated plates and incubated for 2 to 4 days at 37°C in a CO_2_ incubator. Following this, TSOs were treated with vehicle, docetaxel, selumetinib, anti-IL-6 Ab, anti-G-CSF Ab, or combinations along with Click-IT EdU reagent (final concentration 1 μm) for an additional 3 days. At the end of treatment, media was carefully removed, washed with PBS, and fixed with 4% paraformaldehyde in PBS for 30 min at 37°C. Fixative was removed and cells were washed with 3% BSA in PBS, followed by permeabilization with 0.5% Triton X-100 for 30 min. This was followed by washing and incubation with 1× Click-IT reaction mixture for 30 min at room temperature and washing and counterstaining with Hoechst 33342 (1:2,000 dilution) for 20 min and imaging using an ECHO Revolve fluorescence microscope.

### FUCCI imaging

D2.0R-FUCCI cells, 2H11s, or MEFs, were coated with nanoshuttle beads the day before spheroid formation. On the day of spheroid formations, D2.0R-FUCCI cells, 2H11 and/or MEFs were cultured as monotypic, double, or TSS with D2.0R-FUCCI: 2H11: MEF in the ratio of 1:2:2 in DMEM with low glucose and 2% FBS for 3 to 4 days in a CO_2_ incubator. Then, media was replaced with fresh media containing vehicle, docetaxel, anti-IL-6, anti-G-CSF, selumetinib, or combinations. These cells were imaged using multichannel Incucyte Live Imaging system or ECHO Revolve.

### Cell viability assay

Nano shuttle-coated D2.0R cells, 2H11 cells, or MEF cells were cultured for 3 to 4 days in DMEM + 5% FBS, seeded at 5,000 cells/well in a 96-well plate to allow spheroid formation with help of a driver magnetic base. Spheroids were then treated with different doses of docetaxel (0 to 10 μm) in fresh culture medium. At the end of treatment, plate was equilibrated at room temperature for 30 min and 100 μl CellTitre Glo 3D (Promega) cell viability assay reagent was added to 100 μl of cells in the well with vigorous shaking for 5 min. Cells were incubated for 25 min at room temperature and luminescence was recorded using Clariostar Plus microplate reader.

### Cell invasion assay

D2.0R cells were labeled with CellTracker Deep Red dye and suspended in chemotaxis buffer (DMEM containing 0.1% BSA + 2% RGF BME). CellTracker labeled D2.0R cells were mixed with 2H11 and MEF cells in the ratio of 1:2:2 at 5 ×10^4^ cells/mL in chemotaxis buffer (DMEM + 0.1% BSA + 2% RGF BME). Next, 2.5 × 10^4^ cells were applied to the upper chambers of 8-μm PET Growth Factor Reduced Matrigels (24-well format), 0.5 mL chemotaxis buffer was added in the bottom well and cells were incubated for 1 day at 37°C in a humidified 5% CO_2_ incubator. Cells were treated with 1 μm DTX and incubated at 37°C in a humidified 5% CO_2_ incubator for 2 days. At the end of this, cells were scraped from inside the Matrigel insert; the bottom side was fixed and stained with DAPI. The peeled GFR Matrigel was carefully mounted on a glass slide. Two separate fields of cells were counted for each invasion assay at 10× objective and expressed in terms of total number of invading cells ± S.E.


$$\%\;\mathrm{Cell}\;\mathrm{invation}=\frac{\#\;\mathrm{of}\;\mathrm{Celltracker}\;\mathrm{Deep}\;\mathrm{red}\;\mathrm{positibe}\;\mathrm{cell}\;\mathrm{invaded}}{\mathrm{Total}\;\mathrm{number}\;\mathrm{of}\;\mathrm{cancer}\;\mathrm{cells}\;\mathrm{added}\;\mathrm{in}\;\mathrm{the}\;\mathrm{top}\;\mathrm{insert}}*100$$

### In vivo tumor dormancy model

For orthotopic tumor dormancy, 5 × 10^4^ D2.0R luc-mCherry cells were injected in the fourth mammary fat pad (mfp) of 6 to 8 weeks old immunocompetent Balb/cJ mice. Metastatic tumor dormancy in the lungs was established by injecting D2.0R luc-mCherry cells intravenously (0.5 × 10^6^) in 6 to 8 weeks old immunocompetent Balb/cJ mice. Cell viability was assessed by trypan blue exclusion prior to injection and was always above 90% viability. All mice used in experimentation were 6 to 8 weeks old female Balb/cJ mice and killed by CO_2_ asphyxiation followed by cervical dislocation unless otherwise stated. All procedures were approved by the Emory University Institutional Animal Care and Use Committee (Protocol # PROTO201800020) and conformed to the Guide for the Care and Use of Laboratory Animals.

### Bioluminescence imaging

Mice were injected with 150 mg/kg of D-Luciferin intraperitoneally under isoflurane anesthesia. Mice were imaged using IVIS Spectrum with bioluminescence settings on the Living Image software. The bioluminescence intensity was computed as total flux (p/s) by Living Image software normalized to background bioluminescence.

### Proteomic measurements

Cytokines in the discovery sample set were measured using the BioPlex 200 MD31 Mouse Cytokine Array/Chemokine Array (Eve Technologies, Calgary, AB). Cell culture supernatant or mouse plasma was collected and shipped overnight on dry ice to Eve Technologies for analysis by BioPlex 200 MD31 multiplex immunoassay. Cytokines that were below the lower limit of quantitation were excluded from downstream analyses. Results show the standard error of mean of samples.

### Drug administration

All drugs were administered via intraperitoneal injection except selumetinib, which was given twice daily by oral gavage. Docetaxel (USP) was diluted in 0.9% saline and administered once (day 0) and selumetinib was dissolved in DMSO/PEG/sterile PBS and administered for 8 days starting the day before chemotherapy. For cytokine ablation, mice were treated with 200 μg anti-mouse IL-6 (clone MP5-20F3) once on alternate days and/or 10 μg anti-mouse G-CSF (clone 67604) every day for 8 days starting the day before chemotherapy. Vehicle or a rat IgG1 isotype control (clone HRPN) were administered to mice who did not receive the drugs or cytokine ablation.

### Flow cytometry

For in vitro experiments, TSOs were digested with TrypLE and Accumax and passed through 40 μm strainers to obtain single-cell suspension. For in vivo experiments, fourth mfp were collected from mice at necropsy. To prepare for flow cytometric analysis, mfp was digested in DMEM/F-12 (1:1) media containing collagenase, hyaluronidase, DNAse I, and passed through a 70 μm strainer to obtain a single-cell suspension. Red blood cells were lysed using the ACK lysis buffer. Samples were then incubated with LIVE/DEAD Fixable Aqua Dead Cell Stain. After washing, samples were incubated in the presence of an anti CD16/32 Fc receptor-blocking antibody followed by surface staining of the following antibodies in 3 panels: Panel 1: APC-Cy7 CD3, FITC CD4, PerCP-Cy5.5 CD8, APC CD25; Panel 2: PE-CF594 Ly6G, PerCP-Cy5.5 Ly6C, FITC CD11b, eFluor450 F4/80, Super Bright 645 CD80, and PE-Cy7 CD206; and Panel 3: Alexa Fluor 594 anti-mCherry, Alexa Fluor 647 CD34, FITC Ki67 with the appropriate isotype controls.

FoxP3 or Ki67 staining was performed using the eBioscience FoxP3/Transcription Factor Staining Buffer Set, a PE FoxP3 antibody and a FITC Ki67 antibody. All samples were run on a Cytek Aurora flow cytometer and analyzed using FlowJo software.

### Immunohistochemistry

Tumors/mfps were excised from mice. These tissues were fixed in 10% neutral buffered formalin, embedded in paraffin, sectioned (5 μm), and stained with HE. Immunohistochemical staining was performed by the Cancer Tissue and Pathology Core Lab at Winship Cancer Institute of Emory University.

### Immunofluorescence staining of tumor/mammary tissue

Tumors/mfps were excised, fixed, and processed as mentioned above in the immunohistochemistry section. Slides were deparaffinized and rehydrated by serial xylene and ethanol washes. For antigen retrieval, slides were incubated in 1× citrate buffer (pH 8) in a steam cooker for 15 min. Slides were then brought to room temperature and washed in water 2 times. Slides were blocked with Fc receptor blocker and 1× blocking buffer (5% normal goat serum, 0.1% Triton X-100, and 2.5% BSA in PBS) for 1 h. Sections were then incubated with anti-mCherry (1:100) and anti-phosphoERK (1:100) in 0.5× blocking buffer overnight at 4°C in a moisturized staining chamber. After 3 washes with PBS, sections were incubated with Alexa Fluor 488-conjugated secondary anti-mouse (1:200) for 1 h at RT. After 3 washes with PBS, sections were counterstained with DAPI and rinsed in water, and the slides mounted onto coverslips using mounting media. Stained sections were imaged using an ECHO Revolve microscope.

### Quantification of metastatic burden and metastatic foci

The metastatic burden in lungs was evaluated using hematoxylin and eosin-stained lung sections. Metastatic burden was plotted as area of several tumor foci in the lungs, calculated as percentage of lung area.

### Single-cell RNA sequencing library preparation, sequencing, and analysis

For in vitro experiments, multicellular tumor stromal organoids (TSOs) treated with VEH or DTX were digested with TrypLE and Accumax and passed through 40 μm strainers to obtain single-cell suspension, which was processed using 10× genomics kits. For in vivo experiments, tumors/mfps from control and docetaxel-treated mice at 45 days post-chemotherapy were processed as described in flow cytometry above for preparing single-cell suspension and downstream processing was done using the 10× genomics kits. ScRNA-seq libraries were prepared using the Next GEM Chromium single-cell 3′ reagent kits V3.1 with feature barcode technology for cell surface protein (10× genomics) and sequenced using NextSeq 500 high output kits and Novaseq S4 PE100 kits (Illumina). scRNA-Seq data after standard quality control was aligned to the reference genome (mm10) using the 10× Cell Ranger pipeline. Preprocessed and filtered normalized data were subjected to unsupervised analysis using principal component analysis (PCA) (Seurat v2.0 Bioconductor package [15]) to identify principal components with significant variation that was used as input for UMAP, Uniform Manifold Approximation and Projection (UMAP) analysis to determine overall relationship among cells. Cells with similar transcriptome profiles were clustered together, and the clusters were subsequently annotated to different cell types based on expression of specific transcripts, e.g., Endothelial cells (*Cd34+*, *Eng+*, *Pecam1+*), Fibroblasts/CAFs (*Fbn1+*, *Cd34+*, *Pdpn+*), cancer cells (*Krt18+*, *Krt8+*). Transcripts significantly associated with a particular cell type were identified by comparing the gene expression profile of the target cell with the rest of the cells using nonparametric Wilcox’s rank test (*P* < 0.01) and fold change (>1.2).

### CellChat analysis

Cell–cell communication analysis was performed using Cellchat [16] individually on the dormant and DTX treated groups and the mergeCellChat() function was used to compare them. Single-cell data was input as a normalized count matrix containing the expression of all the genes present in the dataset that helped uncover the underlying pathways associated with the 2 groups of analyses.

### SDS-PAGE and western blotting

To confirm the MEK activity, we measured levels of MEK1/2, ERK1/2, and phospho-ERK1/2 proteins in D2.0R cancer spheroids treated with conditioned media (CM) from stromal spheroids and performed SDS-PAGE and western blot analyses specific for each of these 3 proteins, as well as using GAPDH as the equal protein loading control. Approximately 50 μg of protein lysate (lysis buffer composition was: 50 mM HEPES (pH 7.2), 100 μm Na_3_VO_4_, 0.1% Triton X-100, and 1 mg/mL each of protease inhibitors (aprotonin and leupeptin) was loaded per each lane of the SDS-gels that were then used for western blot analyses. Since the molecular weight of the proteins of interest were in the 40 to 45 kDa range, we ran 2 gels simultaneously with equal protein loading to avoid stripping and re-probing the blots several times. For western blot analyses, mouse phospho-ERK1/2 (clone E10) IgG, rabbit ERK1/2 IgG, rabbit MEK1/2 IgG (clone 47E6), rabbit GAPDH IgG (clone 14C10), and rabbit Histone H3 IgG (clone D1H2) were utilized as primary antibodies according to the manufacturers’ recommendations. Anti-rabbit or -mouse IgG HRP antibodies were used as secondary antibodies. Immunoreactivities were detected using SuperSignal West Femto chemiluminescence substrate (ECL) reagents from Thermo Fisher and Bio-Rad Chemidoc XRS+.

### Statistics

Statistical analyses were performed as described in individual figure legends. Generally, *P* < 0.05 was considered significant and statistical tests for in vitro and in vivo experiments were two-tailed, unless otherwise indicated. Statistics for transcripts significantly associated with a particular cell type in the single-cell RNAseq data were identified by comparing the gene expression profile of the target cell with the rest of the cells using nonparametric Wilcox’s rank test (*P* < 0.01) and fold change (>1.2). For flow cytometry analyses, statistical significance were conducted with one-way analysis of variance (ANOVA) with Brown–Forsythe F-test followed by Dunnett’s multiple comparisons post hoc test for comparing different treatment groups, unless otherwise indicated. For in vivo experiments, independent *t* test and one-way ANOVA with Dunnett’s post hoc comparisons were utilized. Independent *t* test was utilized to evaluate significance in in vitro experiments with less than 3 treatment conditions. The Kolmogorov–Smirnov test was used to evaluate the assumption of normality of continuous variables, and no significant departures from normality were detected. When applicable, data distribution was assumed to be normal. Summary data are reported as mean ± SEM. Longitudinal luminescence intensity data were analyzed using ordinary two-way ANOVA followed by Dunnett’s post hoc comparisons between different treatment groups.

Biological replicates for each experiment are noted in figure legends. No data were excluded from the analyses. Mice were randomized from different cages and allocated to vehicle and treatment groups for all in vivo experiments. Immunohistochemistry images were acquired and analyzed in a blinded fashion. For all other experiments, neither randomization nor blinding was used.

## Results

### Chemotherapy breaks cancer cells out of dormancy and induces cancer proliferation and invasiveness

**Fig 1:**
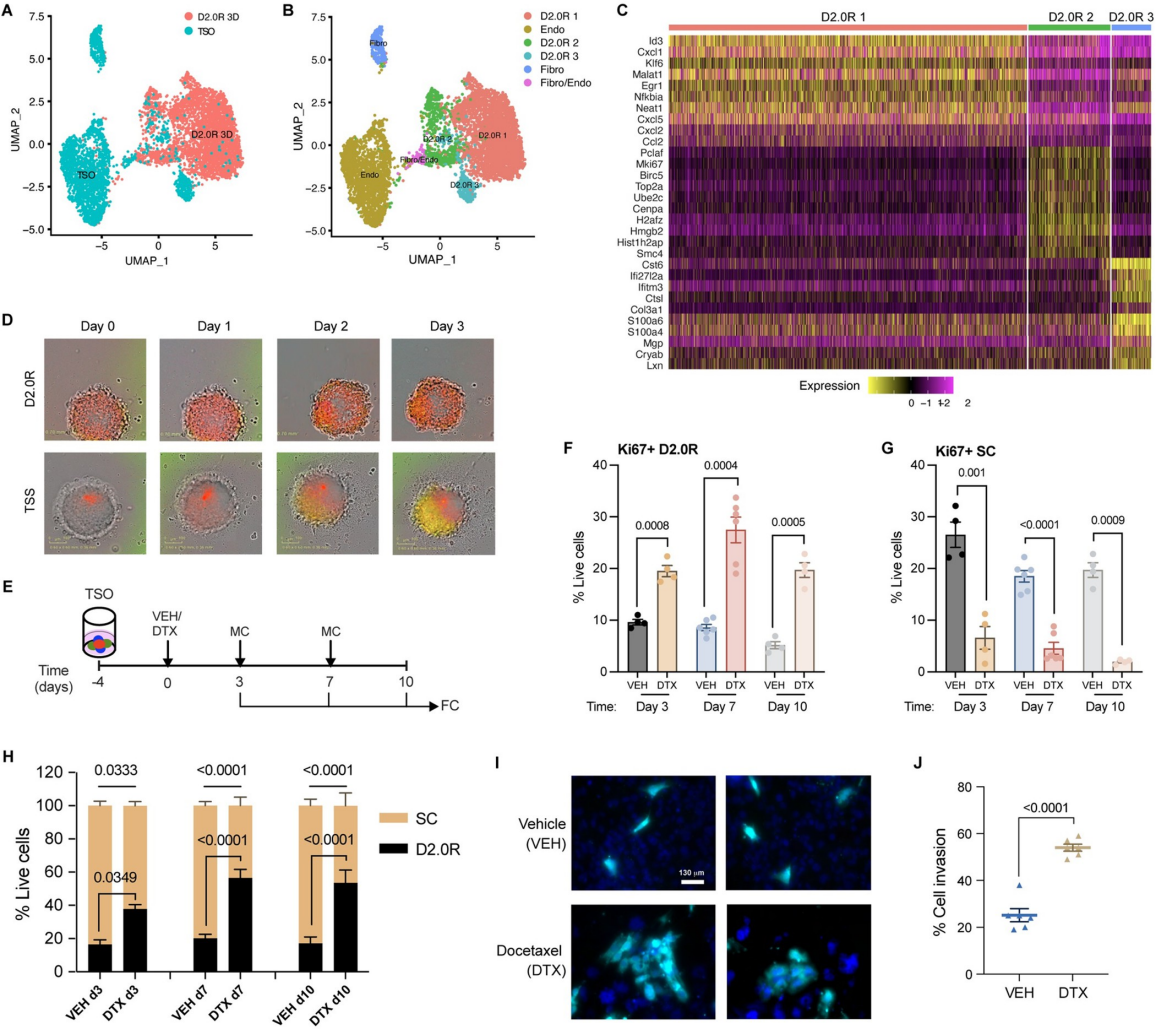
Docetaxel invokes dormancy outbreak in TSO and promotes invasion. **(A)** UMAP presentation of monotypic 3D culture (D2.0R 3D) (*n* = 24) and TSOs (*n* = 24) from scRNA-SEQ analysis. **(B)** UMAP presentation of major cell type clusters from scRNA-SEQ analysis in a merged dataset. **(C)** Heatmap showing the top 10 DEGs in each of the cancer cell clusters in a merged dataset. **(D)** Representative FUCCI reporter images of cancer spheroids (D2.0R) and TSSs (D2.0R-FUCCI: Endothelial cells (ECs): Fibroblasts (Fibro)) upon docetaxel treatment (*n* = 3); scale 360 μm^2^. **(E)** Experimental design used to test the effects of chemotherapy on TSOs; VEH—Vehicle, DTX—Docetaxel, MC—Media change, FC—Flow cytometry. **(F, G)** Expression (%) of Ki67 in **(F)** cancer cells (D2.0Rs) and **(G)** stromal cells (ECs and Fibroblasts) in TSO upon docetaxel (DTX) treatment compared with vehicle treated control on days 3 (*n* = 4), 7 (*n* = 6), and 10 (*n* = 4) from the start of treatment. Independent *t* test measurement shows statistical significance between treatment groups. **(H)** Stacked bar graph showing percentage of cancer cells and stromal cells out of total live cells in vehicle and docetaxel-treated TSO on days 3 (*n* = 4), 7 (*n* = 6), and 10 (*n* = 4). Independent *t* test measurement shows statistical significance between treatment groups. **(I)** Representative images of CellTracker Deep Red dye labeled cancer cells (cyan) that invaded through the Matrigel upon vehicle or docetaxel treatment with DAPI (blue) staining for nuclei. **(J)** Quantification of total number of cancer cells (cyan) invaded through the matrix relative to total cells added per well (%), *n* = 3 independent experiments in duplicates. Independent *t* test measurement shows statistical significance between treatment groups. Flow gating strategy for this figure can be found in S1 Fig and raw flow cytometry data is available on Flow repository (FR-FCM-Z6HQ). scRNA-seq source data is available on GEO (accession # GSE231350). Source data and source code can be found in S1 Data and S1 Code, respectively. DTX, docetaxel; FUCCI, fluorescence ubiquitination cell cycle indicator; scRNA-seq, single-cell RNA sequencing, TSO, tumor stromal organoid; TSS, tumor stromal spheroid; UMAP, Uniform Manifold Approximation and Projection.

To investigate the effects of chemotherapy on tumor mass dormancy in the tumor stromal environment, we extended a well-characterized model of cancer dormancy using D2.0R breast cancer cells [5,6] into a physiologically relevant in vitro TSO model comprising cancer cells, fibroblasts, and endothelial cells as described in Materials and methods. To establish this model, we first characterized the traditional monotypic 3D culture (D2.0R 3D) [6] and our extended TSO culture (TSO) using droplet based single-cell RNAseq analysis showing the transcriptome profiles of 3370 and 2375 individual cells from D2.0R 3D and TSO cultures, respectively (Fig 1A). A combined UMAP cluster map and heatmap show the relative abundance and transcriptome profiles of cell types in the monotypic D2.0R 3D culture and the TSO culture (Figs 1B and S1A). To understand the transcriptome profiles of cancer cells in monotypic versus TSO culture, we subset out cancer cell clusters (i.e., D2.0R 1, D2.0R 2, and D2.0R 3) and noticed that cluster D2.0R 1 was predominantly present in the D2.0R 3D culture (monotypic), whereas cluster D2.0R 3 was predominantly present in the TSO culture. The only cluster commonly present in both the culture models was cluster D2.0R 2 (S1B Fig). Furthermore, by differential gene expression analysis (Fig 1C), we observed that the transcriptomic profile of cancer cells in the D2.0R 3D (i.e., cluster D2.0R 1) shared very few similarities with those in the TSO culture (i.e., cluster D2.0R 3). The cluster that is common to both culture systems (i.e., cluster D2.0R 2) exhibits cell cycle and proliferation associated genes such as *Top2a*, *Ube2c*, *Mki67*, etc. These findings suggested that even in monotypic 3D (D2.0R only) culture, all the cells do not undergo cellular dormancy. Some cancer cells continue to proliferate as shown by cancer cell cluster D2.0R 2, suggestive of tumor mass dormancy rather than cellular dormancy. Furthermore, by differential gene expression analysis on total cancer cells in the 2 cultures, we identified several dormancy-associated genes [17] such as *Cst6*, *Mgp*, *Mme*, *Gas6*, etc. to be up-regulated in the TSO culture compared to monotypic culture (S1 Table). Thus, after characterizing and finding tumor mass dormancy by transcriptomics analysis in the TSO culture, we set out to study the effects of chemotherapy on dormancy.

Using physiologically relevant concentrations of docetaxel (0.01 to 10 μm), we performed a dose response cell viability assay to determine the optimal concentration of docetaxel for our study. We observed that even at the highest concentration (10 μm) of docetaxel, cancer cell viability was unaffected, whereas concentrations as low as 1 μm reduced stromal cell viability significantly (S1C–S1E Fig). Using D2.0R cells expressing the fluorescence ubiquitination cell cycle indicator (FUCCI) reporter, we monitored cancer dormancy or cell cycle arrest (noted by red cells) and cancer cell proliferation or cell cycle re-entry (noted by orange-yellow-green cells). We observed that when cancer spheroids (comprising cancer cells alone) are treated with vehicle (VEH) or 1 μm docetaxel (DTX), cancer cells continue to remain in a state of cell cycle arrest or dormancy (noted by red cells) (Fig 1D, top row), whereas docetaxel treatment of TSO (comprising cancer cells and stromal cells) induced cell cycle re-entry in cancer cells (noted by orange-yellow-green cells) (Fig 1D, bottom row). To quantitatively measure cell proliferation or tumor dormancy escape, we performed flow cytometry analysis to determine the percentage of Ki67 expressing D2.0R cells (Flow gating shown in S1F Fig). We observed that following docetaxel treatment, there was a significant increase in the percentage of cancer cells expressing Ki67, while there was a decrease in stromal cells expressing Ki67 (Fig 1E–1G). We also noticed that the increase in proliferation corresponds to an increase in the percentage of total D2.0R cells in the TSO (Fig 1H). Previously, it has been shown that cancer dormancy escape resulted in increased cancer invasion and metastasis [18]. Thus, we investigated if DTX treatment impacts cancer cell invasion. We treated D2.0Rs, cocultured with fibroblasts and endothelial cells in a trans-well insert (coated with RGF Matrigel), with DTX or VEH. We observed an increase in the number of cancer cells that invaded through the Matrigel following DTX treatment compared to VEH treatment (Fig 1I and 1J). These findings suggest that chemotherapy not only causes low proliferative or slow cycling cancer cells to become highly proliferative, but also increases their invasiveness.

### Stromal-derived cytokines drive cancer dormancy escape

**Fig 2:**
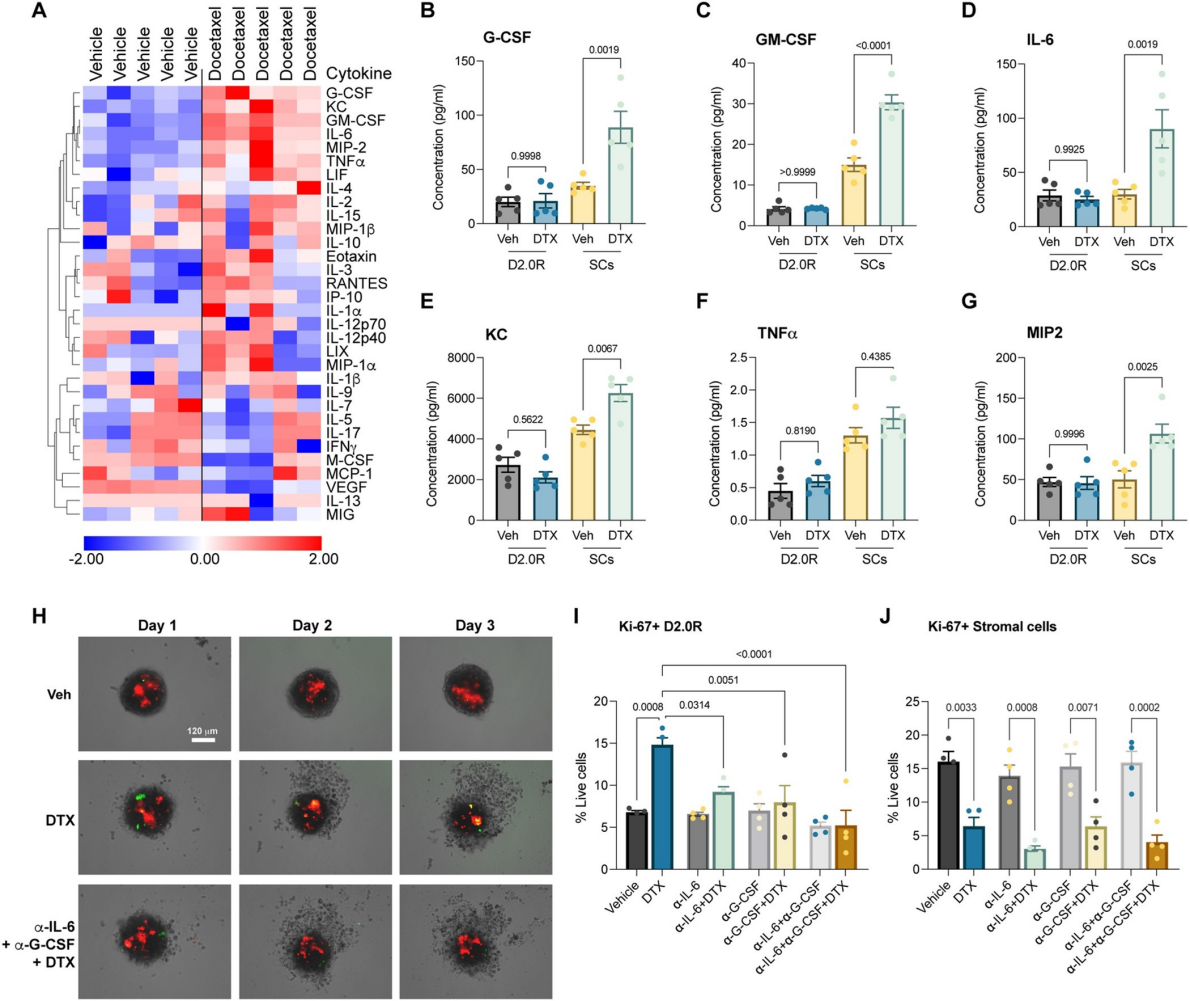
Multiplex secretory protein analysis shows chemotherapy injures stromal cells releasing proinflammatory mediators that invoke cancer dormancy awakening. **(A)** 32-plex cytokine analysis of TSO culture supernatant upon vehicle or docetaxel treatment (*n* = 5). **(B–G)** Concentrations of G-CSF **(B)**, GM-CSF **(C)**, IL-6 **(D)**, KC **(E)**, TNFα **(F)**, MIP2 **(G)** in culture supernatants of cancer spheroids or stromal spheroids treated with vehicle or docetaxel (*n* = 5, each). One-way ANOVA measurement with post hoc Tukey’s multiple comparison test for flow cytometric analysis shows statistical significance between respective treatment groups. **(H)** Representative FUCCI reporter images of TSSs (D2.0R: ECs: Fibro) upon vehicle (Veh), docetaxel (DTX), or cytokine blockade with docetaxel treatment (anti-IL-6+anti-G-CSF+DTX) (*n* = 3); scale, 120 μm. **(I, J)** Expression (%) of Ki67 in **(I)** D2.0Rs and **(J)** stromal cells in TSO upon docetaxel treatment compared to vehicle treated control or cytokine blockade with or without docetaxel treatment (*n* = 4), with 6 replicated per experiment. One-way ANOVA measurement with post hoc Dunnett’s multiple comparison test for flow cytometric analysis shows statistical significance between respective treatment groups. Source data can be found in S1 Data. DTX, docetaxel; FUCCI, fluorescence ubiquitination cell cycle indicator; G-CSF, granulocyte colony stimulating factor; TSO, tumor stromal organoid; TSS, tumor stromal spheroid.

Having noticed that stromal cells are impacted by chemotherapy while cancer cells remain intact, we set forth to determine if stromal injury by chemotherapy and release of secretory factors was causing cancer dormancy escape. To investigate this, we utilized multiplex cytokine assay to determine levels of 32 secretory cytokines, chemokines, and growth factors in the culture supernatant. Interestingly, we found that G-CSF (*P* = 0.0468), GM-CSF (*P* = 0.0007), IL-6 (*P* = 0.0149), KC (*P* = 0.0104), MIP-2 (*P* = 0.0079), and TNFα (*P* = 0.0212) were significantly elevated in the TSO supernatant upon docetaxel treatment (Fig 2A). We also observed that VEGF levels were significantly decreased after chemotherapy (Fig 2A). To identify the source of these secretory factors, we subjected cancer spheroids (comprising of only cancer cells) and stromal spheroids (comprising of only stromal cells) to docetaxel treatment and found that the stromal cells were releasing these chemokines, cytokines, and growth factors (Fig 2B–2G). Based on our findings (Fig 2A–2G) and taking into consideration previous studies showing the protumor effects of IL-6 and G-CSF [19], we further investigated the role of these proinflammatory mediators in cancer dormancy escape. We found that neutralizing antibodies against IL-6 and G-CSF starting 2 days prior to docetaxel treatment significantly inhibited chemotherapy-induced cancer cell proliferation, as shown by fewer yellow-green cells (Fig 2H). We also quantified this by flow cytometry staining and analysis (Fig 2I and 2J). We observed that treatment with neutralizing antibodies against IL-6 and/or G-CSF in vehicle treated wells did not have any effect on proliferation, whereas neutralizing antibodies against IL-6 and/or G-CSF in docetaxel-treated wells inhibited cancer cell proliferation (Fig 2I), with no rescuing effect on stromal cell proliferation (Fig 2J). These findings confirm that IL-6 and G-CSF are important mediators in causing cancer dormancy escape.

### Transcriptomic analysis revealed involvement of MEK signaling in dormancy awakening

**Fig 3:**
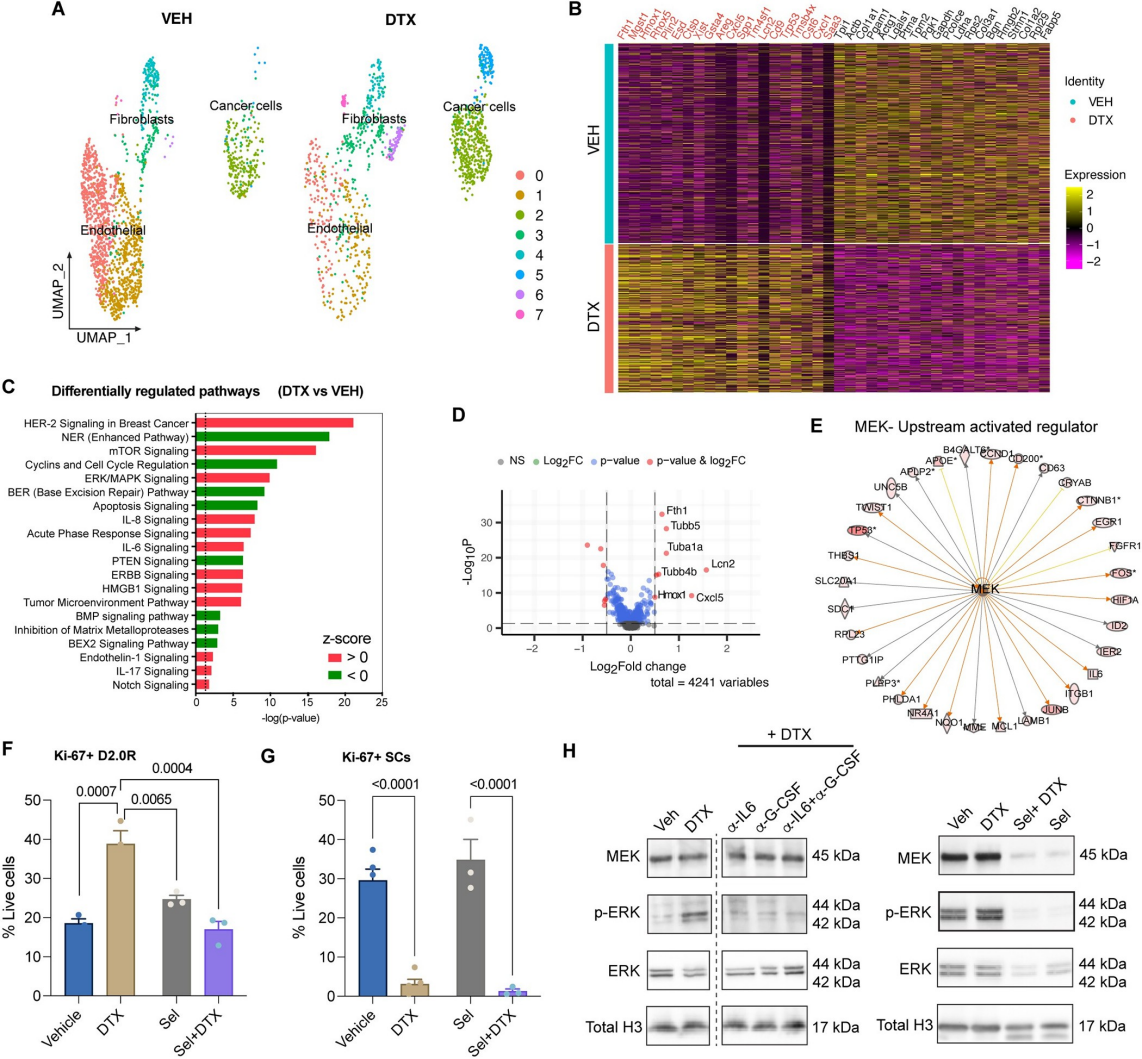
Single-cell transcriptomics revealed MEK signaling in docetaxel-mediated dormancy awakening. **(A)** UMAP presentation of major cell type clusters from scRNA-SEQ analysis of vehicle (VEH) (*n* = 24) and docetaxel (DTX) (*n* = 36) treated TSOs. **(B)** Heatmap showing top 20 enriched genes in DTX and VEH datasets, respectively. **(C)** Pathways enrichment upon docetaxel treatment compared with vehicle controls based on 1,600 differentially expressed genes. Significance for enrichment is calculated based on Fisher’s exact test for each pathway are indicated on the x-axis (-log *p*-value). Color red or green indicates positive or negative z-score, respectively. **(D)** Volcano plot showing significantly differentially expressed protein-coding genes in cancer cells (D2.0R) based on RNA-seq of TSOs from docetaxel treated compared with vehicle-treated controls. Transcripts with absolute FC > 0.5 and adjusted *P*-value < 0.05 are highlighted in red. **(E)** Targets of MEK enriched in cancer cells. Colored lines indicate relationships between nodes, with orange lines showing enhancement and gold lines showing inhibition of a DEG by MEK. **(F, G)** Flow cytometric analysis of proliferating (Ki67+) cancer cells **(G)** and stromal cells **(H)** upon vehicle (*n* = 5), DTX (*n* = 5), selumetinib (*n* = 3) +/- docetaxel (*n* = 3) treatments. One-way ANOVA measurement with post hoc Dunnett’s multiple comparison test for flow cytometric analysis shows statistical significance between respective treatment groups. **(H)** Representative western blot images showing MEK, p-ERK, ERK, and Total H3 (loading control). Raw blot images can be found in S2 Raw Image. scRNA-seq source data is available on GEO (accession # GSE231350). Source data and source code can be found in S1 Data and S1 Code, respectively. DTX, docetaxel; scRNA-seq, single-cell RNA sequencing, TSO, tumor stromal organoid; UMAP, Uniform Manifold Approximation and Projection.

To further investigate molecular signaling and pathways involved in cancer dormancy escape induced by chemotherapy, we performed single-cell transcriptomic analysis. We utilized droplet-based single-cell RNA sequencing (scRNA-Seq) assay to obtain single-cell resolution transcriptomes of TSOs. The transcriptomic profiles of 1738 and 1293 individual cells derived from vehicle and docetaxel-treated organoids, respectively, revealed that docetaxel modulated the tumor stromal landscape by altering the relative abundance (Fig 3A) and transcriptome profiles of cancer cells, fibroblasts, and endothelial cells (S2A and S2B Fig). We observed by split UMAP cluster analysis that the relative abundance of cancer cells was increased in the docetaxel-treated samples (Fold-Change = 2.1), while that of endothelial cells and fibroblasts was decreased (Fig 3A). The differential expression analysis identified 1,600 genes were differentially regulated in docetaxel treatment relative to vehicle control, including 734 overexpressed and 866 reduced genes (*p*-value <0.05) (S2B Fig). Fig 3B shows the top 20 up-regulated genes in DTX and VEH organoid datasets, wherein genes associated with autophagy induced dormancy (*Col1a1*, *Stmn1*) and tumor suppression (*Tpi2*, *Tpm2*) were enriched in VEH group [20–22], while genes associated with chemoresistance (*Fth1*, *Hmox1*), breast cancer tumorigenesis (*Lcn2*), cancer proliferation, and invasiveness (*Cxcl5*, *Spp1*) were enriched in DTX group [23–27]. Ingenuity pathways analysis (IPA) of pathways associated with DTX treatment enriched transcripts demonstrated activation (z-score > 1) of multiple pathways related to cancer pathogenesis, cytokine signaling, cell proliferation, and anti-apoptosis, etc. (Fig 3C). After sub-setting cancer cells (clusters 2 and 5) alone and comparing docetaxel treated to vehicle control, we found 86 genes were differentially regulated with 30 overexpressed and 56 down-regulated (*p*-value <0.05) in the docetaxel-treated D2.0R cells (Fig 3D). The most significantly enriched genes in D2.0Rs upon docetaxel treatment were known protumor genes such as *Fth1*, *Lcn2*, *Cxcl5*, *Tubb5*, *Tuba1a*, *Tubb4b*, and *Hmox1*, which have been implicated in chemo-resistance [23,28,29], breast cancer tumorigenesis [25], invasiveness, metastatic colonization [26], and cancer cell survival [24]. IPA revealed several upstream activated regulatory molecules in cancer cells including *Hras*, *Myc*, *Mek*, etc. Interestingly, several *Mek* (activation z-score = 2.2872, *p*-value = 1.20 × 10^−16^) related genes were seen to be up-regulated in cancer cells as shown in Fig 3E, which play a role in governing cell proliferation.

To confirm the source of *Il6* and *Csf3*, we looked at their gene expression in the TSOs and noticed *Il6* gene expression in clusters D2.0R 2 and Fibro/CAFs 1, while *Csf3* gene expression was only seen in Fibro/CAFs 1 (S2C Fig). Further analysis by treatment revealed that both *Il6* and *Csf3* gene expression increased in Fibro/CAFs 1 after chemotherapy, while there was a marginal decrease in expression of *Il6* in cancer cells (D2.0R 2) with no *Csf3* expression (S2D Fig). It is widely known that MEK/MAPKK is a signaling mediator in the cytokine signaling pathway. Taken together, these findings are in accordance with the protein analysis data from multiplex cytokine assay (Fig 2) implicating a role of stromal injury-mediated paracrine cytokine signaling in cancer cell proliferation via MEK or the ERK/MAPK pathway. To confirm this, we treated cancer spheroids cultured in RGF Matrigel with CM from stromal cells treated with vehicle or chemotherapy. As expected, we found an increase in MEK activity in cancer cells evidenced by no change in MEK or total ERK1/2 levels, but a robust increase in phosphorylated ERK1/2 protein levels in D2.0R spheroids upon chemo-CM treatment (S2E Fig). To confirm the role of MEK in chemo-mediated dormancy awakening, we treated the TSO with selumetinib, a MEK1/2 inhibitor. We found that treatment with selumetinib starting 1 day prior to chemotherapy prevented docetaxel-induced cancer dormancy escape but did not affect stromal cell killing, as shown by a significant decrease in the cancer cell proliferation (i.e., fewer Ki67+ D2.0Rs) and no rescuing effect of stromal cell proliferation (Fig 3F and 3G). We also evaluated cancer dormancy versus proliferation by EdU incorporation assay and found that cancer cells did not show EdU incorporation in vehicle-treated wells, thus confirming dormancy, while we observed EdU incorporation in cancer cells upon docetaxel treatment, which was reversed by cytokine neutralization or selumetinib treatment in the docetaxel-treated wells (S2F Fig). Furthermore, to confirm the effects of cytokine neutralization or MEK inhibitor selumetinib on MEK activity, i.e., phosphorylation of ERK protein, we treated cancer spheroids cultured in RGF Matrigel with CM from stromal cells treated with vehicle (CM-V) or chemotherapy (CM-DTX) and added neutralizing antibodies against IL-6 and/or G-CSF or treated with selumetinib. We observed a decrease in phospho-ERK protein levels with cytokine neutralization or selumetinib treatment in the CM-DTX treated cancer spheroids (Fig 3H). These findings showed that docetaxel-mediated stromal injury and cytokine release activates MEK/ERK signaling in cancer cells resulting in cancer dormancy awakening and that MEK/ERK signaling blockade prevents cancer dormancy outgrowth.

### Docetaxel invokes cancer dormancy escape in vivo

**Fig 4:**
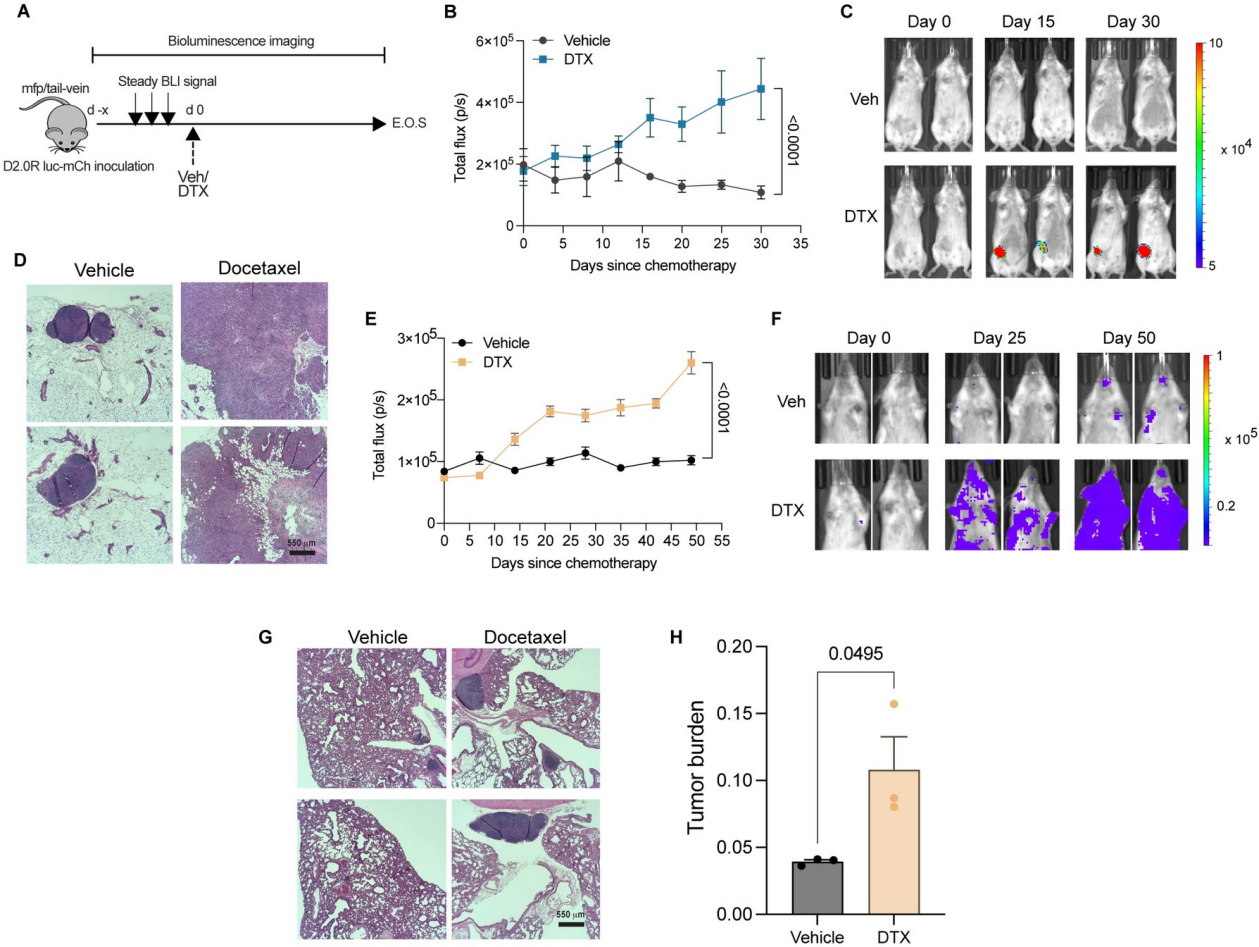
In vivo breast cancer dormancy and docetaxel-mediated dormancy outgrowth. **(A)** Schematic showing mouse model of primary (breast) or metastatic (lung) dormancy by injection of D2.0R luc-mCherry cells in the fourth right inguinal mfp or tail-vein, respectively; days since chemotherapy (d), docetaxel 8 mg/kg (DTX), end of study (E.O.S.), and vehicle (Veh). **(B, C)** Bioluminescence flux kinetics **(B)** and representative BLI images **(C)** of D2.0R luc-mCherry tumor growth in the mfp of mice in docetaxel-treated group (*n* = 9) compared with vehicle-treated controls (*n* = 7). Two-way mixed ANOVA with post hoc Dunnett’s multiple comparisons test shows statistical significance between treatment groups. **(D)** Representative gross images of mfps (fourth right inguinal), HE staining are shown from vehicle and docetaxel-treated mice; scale bars, 550 μm. **(E, F)** Bioluminescence flux kinetics **(E)** and representative BLI images (F) of D2.0R luc-mCherry tumor growth in the lungs of mice in docetaxel-treated group (*n* = 5) compared with vehicle-treated controls (*n* = 4). Two-way mixed ANOVA with post hoc Dunnett’s multiple comparisons test shows statistical significance between treatment groups. **(G, H)** Representative gross images of mouse lungs **(G)**, HE staining, and tumor burden quantification **(H)** are shown from vehicle and docetaxel-treated mice; scale bars, 550 μm. Independent *t* test measurement shows statistical significance between treatment groups. Source data can be found in S1 Data. DTX, docetaxel; HE, hematoxylin and eosin; mfp, mammary fat pad.

To determine whether chemotherapy could directly awaken disseminated and/or dormant cancer cells in vivo, we investigated both orthotopic (primary tumor) and metastatic dormancy using a syngeneic murine breast cancer model. To establish orthotopic dormancy, we injected luciferase-mCherry expressing D2.0R cells in the mfp and tracked cancer cells in vivo by bioluminescence imaging (Fig 4A). We allowed the total luminescent flux to reach a steady basal level with no increasing trend. For our study, we considered the state of steady basal luminescence for at least 2 to 3 consecutive reads as dormancy, which was reached around 30 to 40 days post-inoculation (S3A Fig). Once dormancy was established, we treated mice with vehicle or docetaxel and continued measuring cancer luminescence intensity. We observed that following a single dose of chemotherapy, the luminescence intensity increased significantly over time compared to vehicle-treated mice (Fig 4B and 4C). We also confirmed this by hematoxylin and eosin (HE) staining of the mfp at the end of study, wherein a single dose of docetaxel resulted in an increase in the size of tumor lesion in the mammary tissue compared to the vehicle-treated control mice (Fig 4D). A recent review on cancer dormancy has highlighted that one way of targeting dormant cells is by awakening them and then killing the awakened/proliferating cancer cells [30]. Chemotherapy often preferentially targets proliferating cancer cells and is given for several cycles. To investigate if cancer cells awakened from dormancy by a single dose of chemotherapy can be killed by subsequent cycles of chemotherapy, we administered chemotherapy for 3 cycles. We found cancer cells awakened from dormancy after 1 dose of docetaxel did not succumb to subsequent cycles of chemotherapy as evidenced by lack of response after the third cycle of treatment (S3B and S3C Fig).

To evaluate the role of chemotherapy in metastatic dormancy awakening, we employed the lung metastatic dormancy model [5] by tail-vein injection of D2.0R cells expressing luciferase-mCherry followed by vehicle or docetaxel treatment (Fig 4A). As expected, we observed an increase in the luminescent flux following docetaxel administration (Fig 4E and 4F). We also confirmed metastatic dormancy awakening upon docetaxel treatment by HE staining of the lungs as evidenced by an increase in the lung metastatic burden (Fig 4G and 4H). Thus, our findings demonstrate that chemotherapy invoked both primary/orthotopic and metastatic cancer dormancy escape in a mouse model of breast cancer.

### Chemotherapy induced systemic response and altered tumor immune landscape

**Fig 5:**
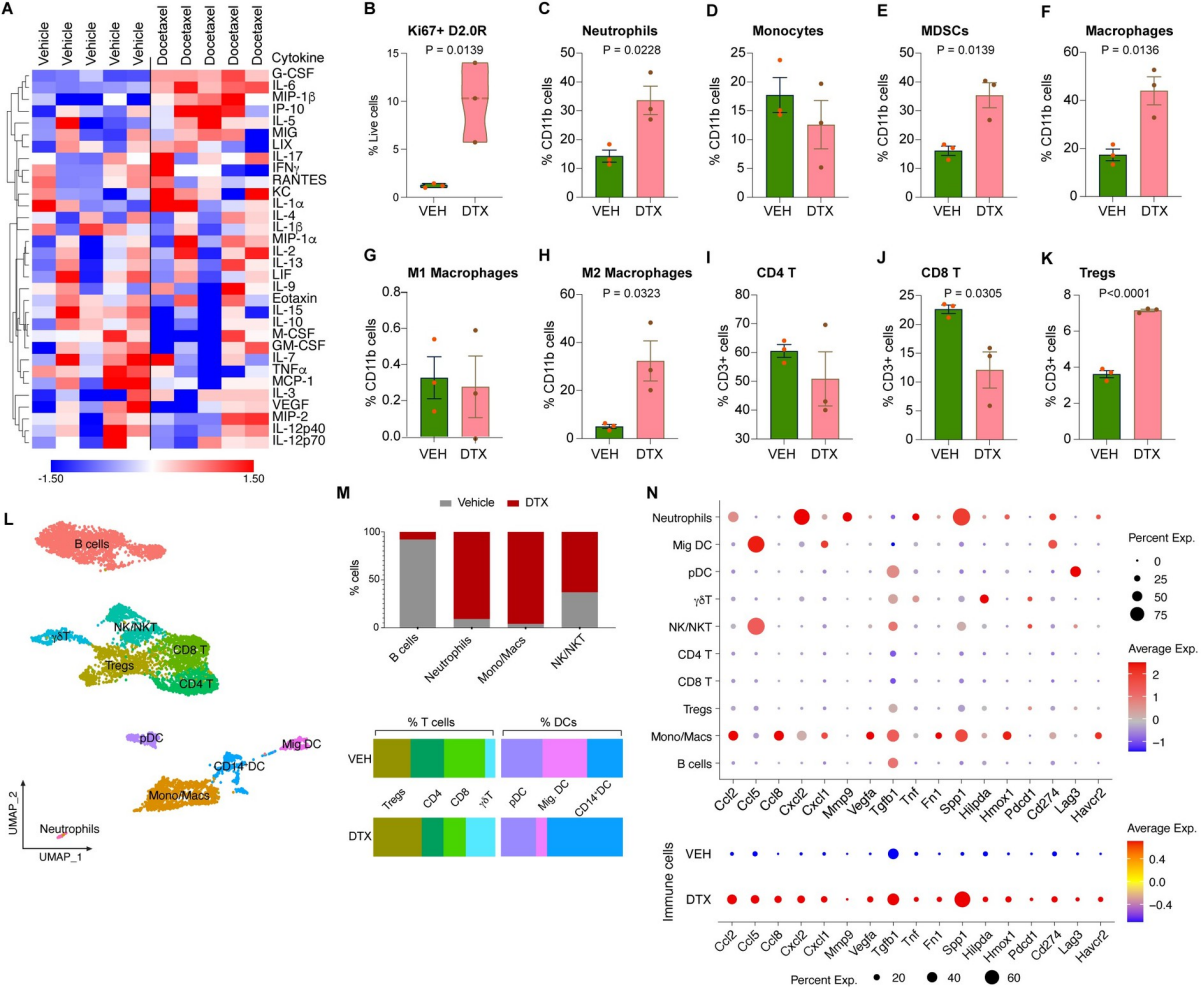
Chemotherapy induced systemic response and altered tumor immune landscape assessment using single-cell transcriptomics, secretory protein, and flow cytometry analyses. **(A)** 32-plex cytokine analysis of plasma from mice subject to vehicle or docetaxel treatment (*n* = 5). **(B)** Expression (%) of Ki67 in D2.0Rs in the mammary tumor tissue of DTX compared to VEH-treated mice. **(C–K)** Flow cytometric analysis showing tumor immune infiltrates including **(C)** neutrophils, **(D)** monocytes, **(E)** MDSCs, **(F)** total macrophages, **(G)** M1 macrophages, **(H)** M2 macrophages, **(I)** CD4 T cells, **(J)** CD8 T cells, and **(K)** Tregs comparing DTX treatment with VEH control (*n* = 3, each). Independent *t* test measurement shows statistical significance between treatment groups. **(L)** UMAP showing immune cells in the tumor tissue of VEH and DTX datasets merged (*n* = 2, each). **(M)** Percentage of cells from VEH control and DTX-treated mice, per cluster for immune cells. **(N)** Dot plots of selected markers from merged samples (top) and grouped by treatment, VEH and DTX (bottom). Dot size indicates the proportion of cells in each cluster expressing a gene and color shading indicates the relative level of gene expression. Flow gating strategy for this figure can be found in S8 Fig and raw flow cytometry data is deposited on Flow repository (FR-FCM-Z6J8). scRNA-seq source data is available on GEO (accession # GSE231350). Source data and source code can be found in S1 Data and S1 Code, respectively. DTX, docetaxel; UMAP, Uniform Manifold Approximation and Projection.

Having confirmed that chemotherapy awakens dormant cancer cells irrespective of the site of dormancy, we investigated if systemic secretory factors played a role in cancer dormancy awakening. For this, we utilized multiplex cytokine assay to determine levels of 32 cell secretory factors and found that IL-6 (*P* = 0.0043) and G-CSF (*P* = 0.0003) levels were robustly increased in murine plasma upon docetaxel treatment compared to vehicle-treated controls (Fig 5A), in agreement with our in vitro data (Fig 2A). Studies have shown that conventional chemotherapy alters the tumor immune landscape [31], while IL-6 and G-CSF induce neutrophil infiltration, modulate effector T cells and promote tumor progression [19,32]. To further evaluate the effects of these secretory factors on cancer cells and leukocyte infiltration in the tumor, we performed flow cytometry staining and analysis (Fig 5B–5J). We observed an altered tumor-immune landscape upon docetaxel treatment. We found that chemotherapy increased cancer cell proliferation (Ki67^+^) (Fig 5B) and resulted in an increase in immunosuppressive myeloid cells like neutrophils, MDSCs, and M2 macrophages, with no changes observed in monocytes or M1 macrophages (Fig 5C–5H). In the lymphoid compartment, we observed an increase in regulatory T cells (Tregs), decrease in anti-tumor CD8+ T cells in the mammary tissue with little to no change in CD4+ T cells (Fig 5I–5K). We also observed an overall immunosuppressive signature in the tumor as shown by a decrease in the ratio of M1:M2 macrophages and CTL:Tregs (S4A and S4B Fig). Furthermore, by leveraging single-cell transcriptomics analysis of the tumor/mammary tissue from mice treated with vehicle or docetaxel (Figs 5L, S5A, and S5B), we revealed an altered tumor immune microenvironment confirming our flow cytometry data. We noticed an increase in the relative abundance of neutrophils, monocytes/macrophages, γδT cells, Tregs, CD14+ regulatory DCs, NK/NKT cells, and a decrease in CD4^+^ and CD8^+^T cells, B cells and effector DC populations (migratory DCs and pDCs) upon docetaxel treatment (Fig 5M). Diving deeper, we investigated whether these tumor immune infiltrates exhibit pro- or anti-tumor phenotype. Markers of immunosuppression, alternative macrophage activation, pro-angiogenic, hypoxia-related, and inhibitory molecules gene signature [33] including Ccl5/RANTES, Ccl8/MCP-2, Cxcl1/CXCL1, Cxcl2/CXCL2, Mmp9/MMP9, Vegfa/VEGF, Tgfb1/TGF-β1, Tnf/TNF-α, Fn1/FN (Fibronectin), Spp1/OPN (Osteopontin), Hilpda/HIG, Hmox1/HO-1, Pdcd1/PD-1, Cd274/PD-L1, Lag3/LAG-3, and Havcr2/TIM-3, were expressed by these immune cells. Comparative analysis between docetaxel treatment versus vehicle control of the protumor gene signature in immune cells demonstrated an up-regulation of this immunosuppressive gene signature upon docetaxel treatment (Fig 5N). These findings confirmed that docetaxel induces systemic release of IL-6, G-CSF, and an altered tumor microenvironment (TME) with an augmented protumor immune gene signature.

### Single-cell RNA-sequencing (ScRNA-seq) revealed stromal injury response invokes cancer dormancy outgrowth

**Fig 6:**
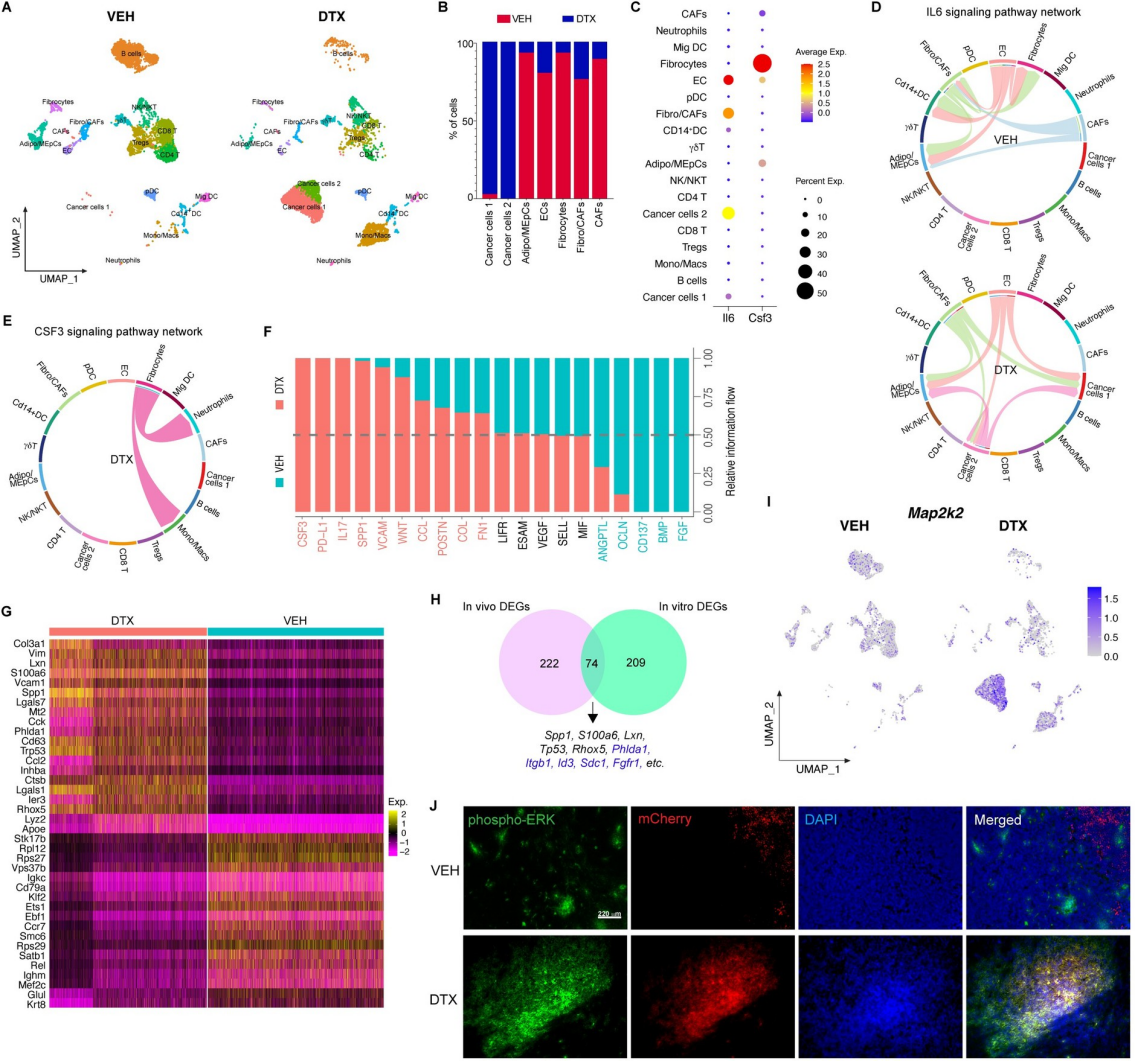
Single-cell RNA sequencing analysis of mouse mammary tumors. **(A)** Split UMAP presentation of major cell types and associated clusters in vehicle (VEH) and docetaxel (DTX)-treated murine mfp/tumor (*n* = 2, each treatment group). **(B)** Percentage of cells from vehicle control and docetaxel-treated mice, per cluster for cancer cells and non-immune stromal cells. **(C)** Dot plot showing cluster wise expression of Il6 (IL-6) and Csf3 (G-CSF) by clusters. **(D)** Chord connections between cell types shows Il6 signaling network in vehicle and docetaxel datasets, respectively. **(E)** Chord connections between cell types showing Csf3 signaling network in DTX dataset. **(F)** Quantitatively comparing the information flow of each signaling pathway between cells from VEH- and DTX-treated samples. The overall information flow of a signaling network is calculated by summarizing all the communication scores in that network. **(G)** Heatmap showing top 20 enriched genes between cells from DTX- and VEH-treated samples, respectively. **(H)** Venn showing cancer cluster DEGs in vivo (pink) and in vitro (turquoise) with overlapping genes at the intersection. **(I)** Split feature plot showing Map2k (MEK) expression in DTX- vs. VEH-treated samples. **(J)** Representative IF images of mfp tumor from VEH- and DTX-treated mice showing immunostaining of phospho-ERK (green), DR.0R-mCherry (red), and nuclei (DAPI); scale bar = 220 μm. scRNA-seq files are accessible on GEO with the accession number GSE231350. Source data and source code can be found in S1 Data and S1 Code, respectively. DTX, docetaxel; mfp, mammary fat pad; UMAP, Uniform Manifold Approximation and Projection.

scRNA-Seq of the mouse mammary tumor/tissue yielded transcriptomic profiles of 6241 and 5587 individual cells from mice treated with vehicle and docetaxel, respectively. Split UMAP cluster maps revealed altered TME with changes in relative abundance and transcriptome profiles of cancer cells, stromal cells, and immune infiltrates (Figs 6A, S5A, and S5B). The analysis depicted the relative abundance of cancer cells and non-immune stromal cells was significantly altered with increased cancer cells and decreased fibroblasts/CAFs, cancer associated fibroblasts (CAFs), fibrocytes, adipocytes/mammary epithelial cells (Adipo/MEpCs) upon docetaxel treatment compared to vehicle-treated control mammary tumor (Fig 6A and 6B). We checked for cell cycle associated gene expression to confirm stromal injury by chemotherapy and noticed down-regulation of S and G2-M phase gene expression in stromal cells upon chemotherapy compared to vehicle-treated controls (S6 Fig). Interestingly, while there was only 1 cancer cell cluster in vehicle-treated dormant tumors (Cancer cells 1), there were 2 cancer cell clusters (Cancer cells 1 and Cancer cells 2) in DTX-treated tumors. A comparative analysis of cell–cell communication based on ligand-receptor expression in the mammary tissue/tumor of vehicle and docetaxel-treated mice was performed using CellChat [16], which quantitatively measures the propensity of cell types to function as a sender or receiver for several major signaling pathways. The analysis showed cancer cell clusters were highly networking in the DTX dataset (shown by blue hotspots), whereas stromal cells were highly networking clusters in VEH dataset (shown by red hotspots) (S5C Fig).

Having found that IL-6 and G-CSF levels were increased in plasma after DTX treatment (as shown in Fig 5A), we asked whether the molecular signaling mechanisms were similar in vivo and in vitro (Figs 2 and 3). To investigate the molecular signaling invoked by IL-6 and G-CSF upon docetaxel treatment, we first identified the source of IL-6 and G-CSF in the TME using single-cell transcriptomics data. We found that the non-immune stromal cells are the primary source of *Il6* (*IL-6*) and *Csf3* (*G-CSF*) in the TME (Fig 6C). Interestingly, we found that the cancer cells (Cancer cells 2) that propagated after DTX treatment also expressed *Il6* gene (Fig 6C). A closer look at the IL-6 signaling pathway network revealed a shift in its role in the dormant VEH-treated versus proliferating DTX-treated TME (Fig 6D). In the dormant TME, IL-6 signaling was constrained to stromal cells such as ECs and fibroblasts which are seen to interact among themselves while in the DTX treated tumor tissue, IL-6 activity occurs primarily between the cancer cells and the ECs, fibroblasts characterizing the emergence of complex interactions between cancer cells and stromal cells upon DTX treatment. IL-6 responsive genes such as *Il17* and *Spp1* [34,35] showed elevated signaling with active communication between cancer cells and immune and non-immune stromal cells in the tumor tissue upon DTX treatment (S5D and S5E Fig). The CSF3 pathway was enriched in the DTX-treated tumor tissue defined by fibrocytes interacting with neutrophils and M2 macrophages while this phenomenon was absent in the dormant state (VEH treated) (Fig 6E). Signaling networks such as VCAM, WNT, FN1, etc., known to be associated with dormancy escape were enriched in the DTX-treated tumor tissue, while BMP and FGF signaling networks, which are known to play a role in cancer dormancy [36,37], were enriched in VEH-treated dormant/control tumor tissue (Fig 6F). Therefore, cell communication analysis provided a comprehensive overview of the transformative cellular signaling in the TME upon treatment with DTX.

We identified the top 20 enriched genes in DTX- and VEH-treated mammary tumor tissue, respectively and found that *Col3a1*, *Spp1*, *Vim*, *S100a6*, *Mt2*, etc., which are genes associated with cancer cell proliferation, invasion, ECM remodeling, and tumor progression were enriched upon DTX treatment confirming a deleterious role of DTX in vivo (Fig 6G) [38–41]. Additionally, we confirmed that *Mki67* gene expression in cancer cells was increased in the DTX-treated tumors relative to vehicle treated control (S5F Fig) concurring our flow cytometry data (shown in Fig 5B). Having observed that the molecular signaling in vitro and in vivo were similar, we asked if the cancer cells also behave similarly. For this, we performed comparison analysis between gene expression profiles of cancer cells clusters from in vitro and in vivo scRNA-Seq datasets. The resultant Venn diagram revealed the intersection of 74 enriched genes (Fig 6H). Of these 74 commonly enriched genes, we found several genes (highlighted in blue) that were previously shown by IPA to be associated with MEK signaling (Fig 3E). Thus, to confirm this, we focused on the expression of *Map2k2* (*MEK*) in the mammary tumor tissue. Interestingly, we found that MEK expression was robustly increased in the cancer cell clusters upon docetaxel treatment (feature plot in Fig 6I). We also confirmed up-regulated MEK signaling in tumor tissues shown by p-ERK immuno-staining (Fig 6J). We also confirmed this by IPA, wherein we found ERK/MAPK signaling was enriched in cancer cells (S5G Fig). Hence, these findings confirmed that docetaxel alters IL-6 and G-CSF signaling by stromal cells in vivo augmenting MAP2K signaling in cancer cells, thus likely awakening cancer dormancy and promoting cell proliferation.

### IL-6 and/or G-CSF signaling inhibition prevents docetaxel induced breast cancer dormancy escape in vivo

**Fig 7:**
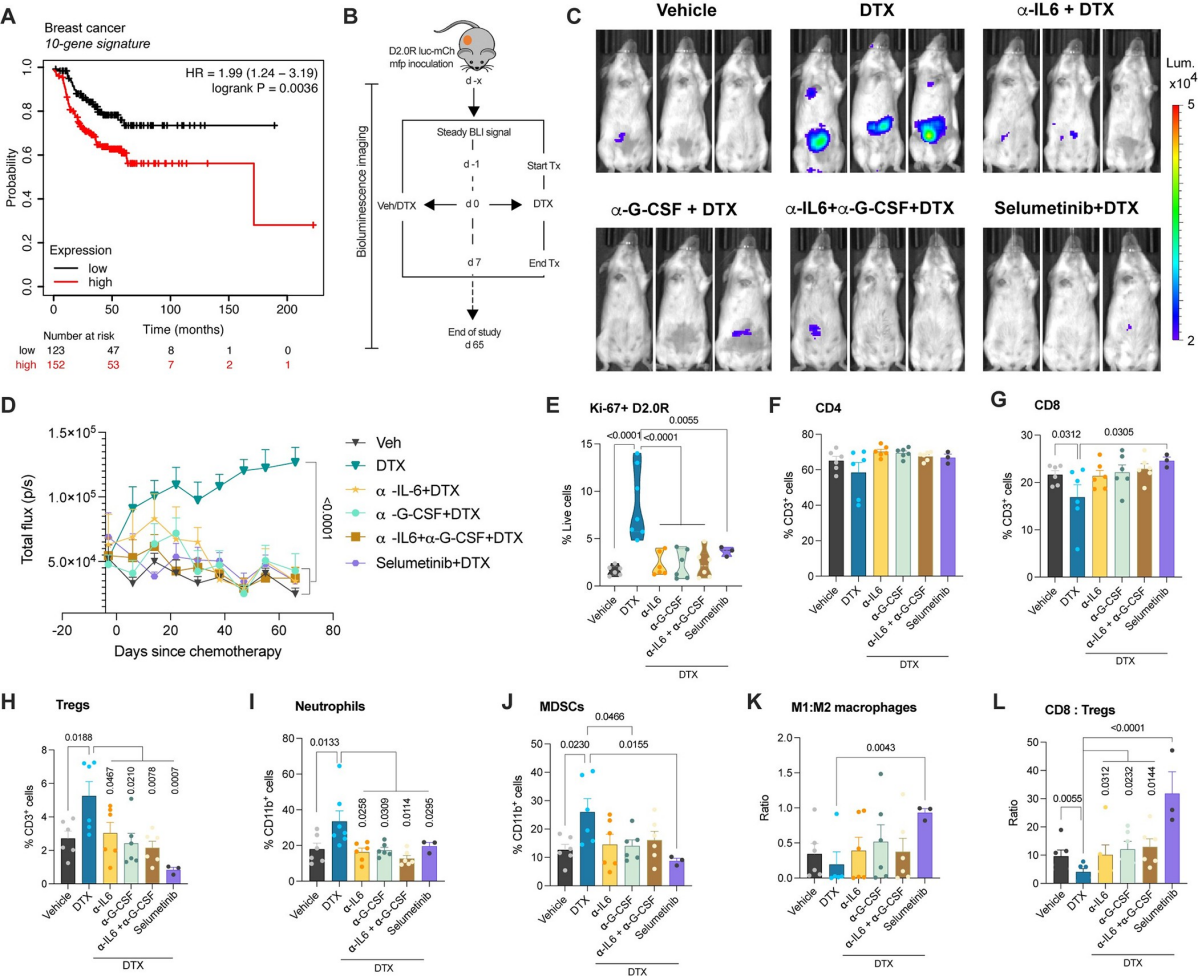
Cytokine ablation and MEK inhibition prevent chemotherapy-induced breast cancer dormancy awakening in mice. **(A)** Kaplan–Meier estimates of recurrence free survival based on a 10-gene signature in breast cancer patients who received chemotherapy. Relevant supporting data can be found in S7 Fig. **(B)** Schematic showing mouse model primary (mfp) dormancy by injection of D2.0R luc-mCherry, treatment groups and schedule; days since chemotherapy (d), docetaxel 8 mg/kg (DTX), neutralizing antibody or MEK inhibitor treatment (Tx) and vehicle (Veh). **(C, D)** Representative bioluminescence images **(C)** and BLI flux kinetics (D) of D2.0R luc-mCherry tumor growth in the mfp of mice in DTX (*n* = 6), α-IL-6+DTX (*n* = 5), α-G-CSF+DTX (*n* = 5), α-IL-6+ α-G-CSF+DTX (*n* = 6), selumetinib+DTX (*n* = 6) compared with vehicle-treated controls (*n* = 6). Two-way mixed ANOVA analysis with post hoc Dunnett’s multiple comparisons test shows statistical significance between treatment groups. **(E)** Expression (%) of Ki67 on D2.0R luc-mCherry cells in mfp of mice from various treatment groups. One-way ANOVA measurement with post hoc Dunnett’s multiple comparisons test shows statistical significance between treatment groups. **(F–J)** Flow cytometric analysis showing percentage of CD4 **(F)**, CD8 **(G)**, Tregs **(H)**, neutrophils **(I)**, MDSCs **(J)** in the mfps/tumors of mice. Independent *t* test or one-way ANOVA measurement analysis with post hoc Dunnett’s multiple comparisons test shows statistical significance between treatment groups. **(K, L)** Ratio of M1:M2 macrophages and CD8:Tregs in the mfps/tumors of mice. Independent *t* test measurement shows statistical significance between treatment groups. Flow gating strategy for this figure can be found in S8 Fig and raw flow cytometry data is deposited on Flow repository (FR-FCM-Z6J8). Source data can be found in S1 Data file. DTX, docetaxel; mfp, mammary fat pad.

To understand the clinical relevance of chemotherapy-mediated dormancy awakening, we used the gene set enriched in chemotherapy-treated samples compared to vehicle-treated controls (genes at the intersection of Fig 6H and S2 Table) in our study. First, we identified which genes from the list of 66 genes contribute to survival in BRCA using the open source GSCA online tool [42]. We found that only 10 genes (APRT, COL18A1, CST6, CTHRC1, HSBP1, ID3, NREP, NUPR1, PTTG1IP, and SDC1) out of the 66 DEGs contributed to survival in BRCA with a hazard ratio (HR > 1) and logrankP-value < 0.05 (S3 Table). The 10-gene set was evaluated by gene set enrichment and gene set variation analyses. Enrichment plot showed a normalized enrichment score (NES) of 1.23 with -log10 (padj) of 0.22 (S7A Fig). GSVA score for the 10 gene set was higher for BRCA tumor compared to normal tissue (*p*-val = 6.09E-43) as shown in S7B Fig. The association between GSVA score and survival was computed (S7C and S7D Fig and S4 Table), demonstrating high GSVA score associates with poor disease specific survival (HR 1.68, *p*-val = 0.02) and shorter disease-free interval or recurrence free survival (HR 1.55, *p*-val = 0.048). Gene set expression changes relevant to subtype and pathologic stages in BRCA (S5 Table) exhibit an upward trend in expression changes in stages III and IV compared to early stages, demonstrated by an upward trend in GSVA score (S7E Fig). However, the GSVA scores did not vary between the different subtypes of breast cancer. Correlation analysis between GSVA score for changes in the gene set and cancer-associated pathways activity showed positive correlation with EMT and RAS/MAPK pathways activation and negative correlation with hormone AR/ER and DNA damage pathways (S7F Fig). Thus to validate this 10-gene signature in predicting recurrence free survival probability in breast cancer, we used the KM-plotter tool [43]. A meta-analysis with the 10-gene signature in 275 breast cancer patients who received systemic chemotherapy alone revealed that high expression of the gene signature correlates with poor recurrence free survival probability (Fig 7A and S6 Table).

After confirming the systemic release of secretory factors (IL-6 and G-CSF), their molecular signaling, alterations to the tumor immune landscape and determining the prognostic importance of the gene signature generated, we next investigated whether inhibiting these cytokines or MEK signaling would prevent cancer dormancy escape. For this, we performed cytokine neutralization or inhibition of the cytokine signaling mediator MEK (using selumetinib) prior to chemotherapy and studied in vivo dormancy versus awakening (Fig 7B). After establishing dormancy, mice were treated with neutralizing antibodies against IL-6 and/or G-CSF, isotype control or selumetinib, starting the day before chemotherapy. We observed that cytokine neutralization with neutralizing antibodies against IL-6 or G-CSF or both significantly inhibited chemotherapy induced dormancy awakening as shown by in vivo luminescence imaging (Fig 7C and 7D). Similarly, we found that selumetinib shown by transcriptomic analysis to mediate the effects of proinflammatory factors in cancer dormancy outgrowth also inhibited the deleterious effects of chemotherapy as evidenced by a significant decrease in luminescent photon flux compared to the docetaxel-treated mice and no significant difference between selumetinib-treated mice and vehicle controls (Fig 7C and 7D). Next, we set out to determine the effects of cytokine ablation or MEK inhibition on the tumor immune landscape. For this, we performed multicolor flow cytometry analysis (Fig 7E–7L) and found that in the presence of neutralizing antibodies against IL-6 and/or G-CSF, or inhibition of MEK, the percentage of Ki67+ cancer cells was significantly reduced suggesting rescue from DTX induced dormancy outgrowth (Fig 7E). We also observed a decrease in the protumor immune infiltrates, such as neutrophils, MDSCs, and Tregs upon cytokine neutralization or MEK inhibition, while an increase in cytotoxic CD8+ T cells was only seen with selumetinib treatment (Fig 7F–7J). We noticed a robust reversal of immunosuppressive signatures shown by an increase in the ratios of M1:M2 TAMs and CD8:Tregs, which are important in predicting OS, pCR, or RFS [44,45], with selumetinib treatment starting prior to DTX treatment (Fig 7K and 7L). These findings confirm the causal role of IL-6 or G-CSF-mediated MEK signaling in cancer dormancy awakening and tumor immunosuppression. Thus, inhibiting the inflammatory mediators (IL-6, G-CSF) or downstream MEK signaling prevented chemotherapy (DTX)-induced breast cancer dormancy awakening and ameliorated a potentially tumor immunosuppressive microenvironment.

## Discussion

Despite tremendous progress made in treatments for cancer over the last 2 decades, cancer dormancy awakening followed by systemic recurrence continues to be a significant clinical issue. Breast cancer recurrence rates have reduced phenomenally with current treatment regimens in clinic; however, there is still 6% to 23% chance of cancer recurrence [46], either locoregional or metastatic, within the first 5 years and about 30% rate of recurrence after 5 years from initial diagnosis and treatment [47,48]. Furthermore, the mean time from distant recurrence to death in ER negative breast cancer patients was less than 3 years [49]. The cause of cancer recurrence varies depending on the type and size of cancer, receptor status, failure to identify local lymph node metastasis, and/or undiagnosable dormant cancer cells.

Recent studies have drawn ample attention to cancer dormancy and its role in cancer recurrence [30,50]. Targeting proliferating cancer cells is the first line of thought in treating cancer, but it is important to address the silent killer within, viz dormant cancer cells. Cancer dormancy could be targeted in a number of ways: (i) cancer dormancy could be prolonged eternally; (ii) dormant cancer cells could be killed without being awakened from dormancy [51]; or (iii) killed after being awakened.

Chemotherapy is given to many cancer patients, as neoadjuvant, adjuvant, or palliative therapy. Some studies have shown that chemotherapeutics induce dormancy in highly proliferating cancer cells [52], while others have shown chemotherapy induces cancer proliferation and metastasis [12,13]. Our study supports the latter view. Using a model of breast cancer dormancy, we showed for the first time that chemotherapy awakens dormant cancer cells by means of stromal injury response without affecting cancer cells directly.

We demonstrate the adverse effects of taxane-based chemotherapeutics as invoking dormant cancer outgrowth. Using a TSO model, we found that these deleterious effects occur in stromal cells that are sensitive to taxane and which release proinflammatory cytokines, such as IL-6 and G-CSF (Figs 1–3). Clinical data from several studies show that taxane therapy increases serum IL-6 and G-CSF levels in patients [53,54] and that higher levels of serum IL-6 is a prognostic marker for early breast cancer recurrence in patients receiving systemic therapy [55,56]. Additionally, IL-6 and G-CSF have been widely implicated in cancer invasiveness [57,58] and recent studies have also shown a role of trans-IL-6 activating JAK/STAT signaling in dormancy awakening [59]. However, the mechanistic role of IL-6 and G-CSF in inducing dormancy escape has not been investigated. By using single-cell transcriptomic and pathway analysis, we found that IL-6 and G-CSF engagement in noncanonical signaling via the ERK/MAPK pathway, involved in fibrosis and wound healing response [60,61], to be crucial in cancer cells outgrowth (Fig 3), instead of the canonical JAK/STAT signaling pathway involved in inflammatory response as shown by previous studies [19,59]. The gene signatures identified in the chemotherapy group agreed with previous studies on dormant-emergent cancer and chemoresistance [20,50]. Furthermore, using a syngeneic mouse model of breast cancer dormancy, both orthotopic and metastatic, we confirmed the deleterious effects of taxane-based chemotherapy in dormancy awakening in vivo, and systemic release of proinflammatory cytokines IL-6 and G-CSF. Using single-cell transcriptomics analysis, we confirmed that the source of IL-6 and G-CSF was indeed fibroblasts/CAFs and fibrocytes, in agreement with our in vitro data and previous findings showing stromal IL-6 plays a role in invoking cancer cell proliferation [11]. Based on CellChat analysis, we found that upon docetaxel treatment, fibroblasts/CAFs have the strongest and highest number of interactions with other cell types in the tumor. We also found that in the docetaxel-treated mice tumor IL-6 signaling pathway was strongly signaling between fibroblasts/CAFs and endothelial cells with cancer cells. Interestingly, we also found that in vivo G-CSF/CSF3 signaling was primarily between fibrocytes and neutrophils or M2 macrophages suggesting robust activation of these suppressive immune cells in the cancer microenvironment, data which was corroborated by increased expression of suppressive cytokine and chemokine genes (Fig 6) strongly suggesting a role of M2 TAMs in chemotherapy-induced protumor signaling [62].

Upon investigating the mammary tumor tissue from docetaxel-treated mice, we found an increase in protumor immune infiltrates including M2 macrophages, MDSCs, Tregs, γδT cells, and an enriched gene signature comprising *Vim*, *Spp1*, *S100a6*, *Ccl2*, and *Lgals1* genes. Previous studies have shown that *Vim*, *Spp1*, and *Ccl2* are IL-6 responsive genes [63–65]. Based on Cellchat analysis, we found that Spp1 signaling was initiated by cancer cells in networking with other cells in the TME. Importantly, in vivo blockade of IL-6 or G-CSF signaling or MEK signaling prevents DTX-induced dormancy escape. Taken together, our findings point to a single signaling mechanism (MEK signaling) by which DTX chemotherapy causes breast cancer dormancy outgrowth (in vitro and in vivo).

In the in vivo model of breast cancer, we noticed that dormant cancer cells were awakened with just a single dose of chemotherapy. Following up on this, we were unable to target the awakened/proliferating cells with subsequent cycles of chemotherapy (Figs 4 and S3). Later, we confirmed by transcriptomic analysis that chemotherapy-mediated cancer dormancy escape induced several survival cues in cancer cells including chemoresistance, EMT, invasiveness, and inflammatory phenotype, evident from the expression of *Cav1*, *Col3a1*, *Col5a1*, *Spp1*, *Fth1*, *Hmox1*, *Il6*, *Cxcl10*, *Cxcl1*, *Ccl2*, etc. Interestingly, our findings showed that chemotherapy not only awakened dormant cancer cells, but also caused clonal propagation of cancer cells, wherein 1 cluster gained chemoresistance phenotype expressing genes such as *Cav1*, *Col3a1*, and *Col5a* [66,67], while the other gained an inflammatory phenotype expressing genes [68,69] such as *Cxcl1*, *Cxcl10*, *Ccl2* (S5H Fig).

There are some caveats to our study. First, the organoid system contains only cancer cells and stromal cells (i.e., fibroblasts and endothelial cells). However, the in vivo mammary TME revealed several immune cells being recruited upon stromal injury are suggestive of potential protumor roles (Figs 5 and 6). Secondly, even though our study revealed a role of taxane chemotherapy in stromal injury-mediated dormancy awakening in vitro and in vivo, nonetheless our model does not address the complexity and heterogeneity of the different populations of cancer cells and the microenvironments within a complex tumor.

**Fig 8:**
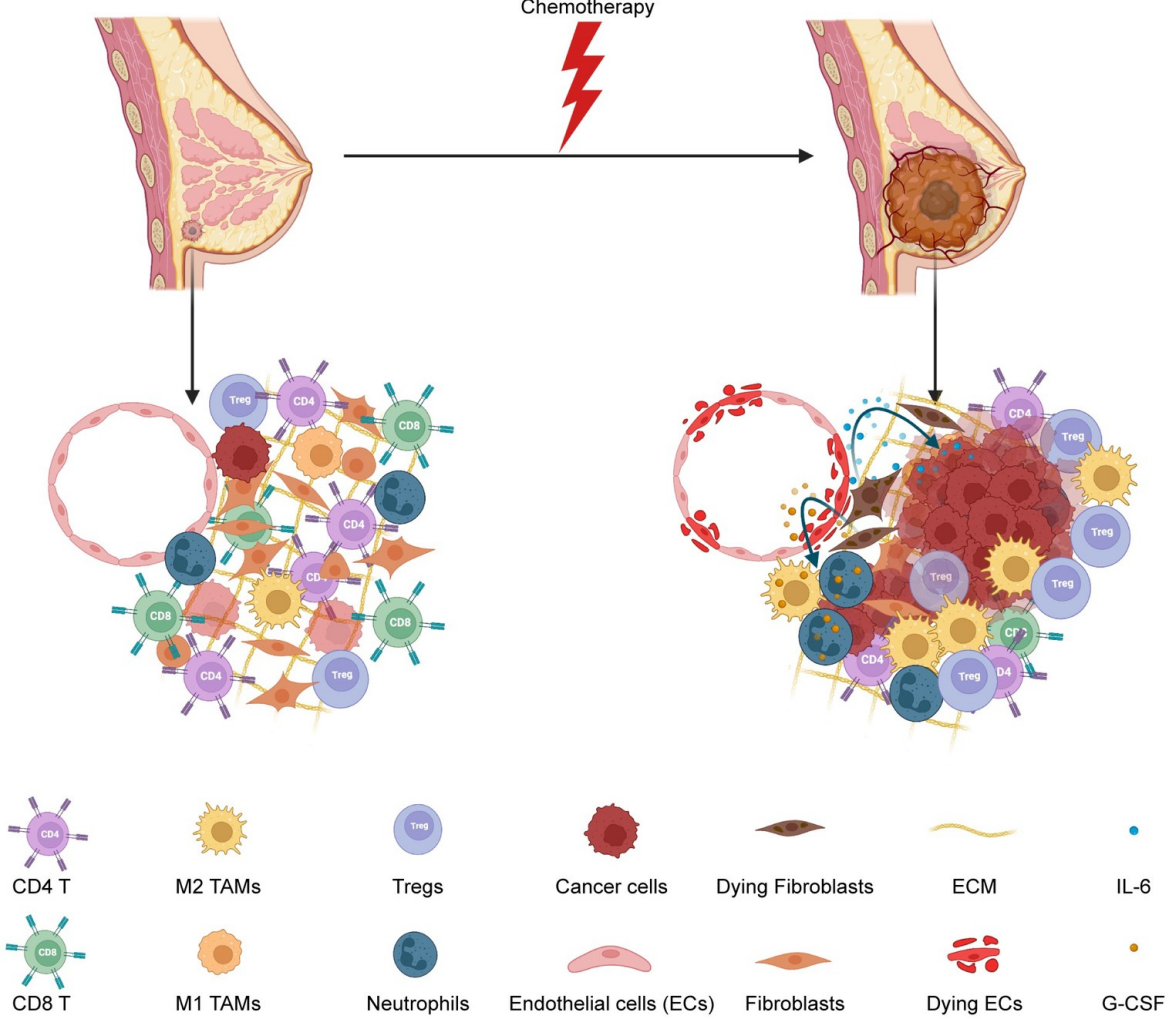
Summary schematic of chemotherapy induced stromal injury and dormancy outgrowth. This schematic shows a dormant tumor in the breast (on the left) with very few dormant cancer cells, healthy stromal cells, and an anti-tumor immune microenvironment. Chemotherapy injures stromal cells releasing IL-6 and G-CSF, which in turn awaken dormant cancer cells, recruit protumor neutrophils, and M2 macrophages. In addition, more protumor immune infiltrates such as Tregs and fewer anti-tumor CD8 T cells were seen after dormancy outgrowth, confirming an overall protumor microenvironment facilitating tumor growth. G-CSF, granulocyte colony stimulating factor.

In conclusion, our study has identified key secretory factors (IL-6 and G-CSF) as mediators of taxane-induced dormancy awakening in a breast cancer model (Fig 8) and a meta-analysis of 10-gene signature predicting recurrence free survival outcomes in patients receiving chemotherapy (Fig 7A). Additional preclinical studies in other tumor types and with other chemotherapies are warranted as are studies in the clinic to monitor changes in the levels of cytokines, chemokines, and growth factors in patients before and after chemotherapy. Our studies suggest that administration of IL-6 or G-CSF signaling inhibitors in the peri-chemotherapy period (at least in patients who might be expected to have a surge of IL-6 or G-CSF levels due to chemotherapy) might decrease tumor recurrence and improve survival outcomes.

## Supporting information

S1 Fig Cell viability assay and flow gating with vehicle and docetaxel treatment. (A) Heatmap showing the top 10 DEGs in each cluster in a merged dataset. (B) Percentage of the different clusters of cancer cells from D2.0R 3D and TSO, per total cancer cells in the respective datasets. (C–E) Dose response curves of cancer cells (D2.0R) (*n* = 5), endothelial (2H11) (*n* = 4), and fibroblasts (MEF) (*n* = 3) to varying concentrations of DTX (0–10 μm). (F) Representative images of flow gating strategy for singlets, live cells, and Ki67+ D2.0Rs (mCherry) (green box) or Ki-67+ stromal cells (2H11:MEF) (red box) in VEH and DTX treated TSOs. scRNA-seq files are accessible on GEO with the accession number GSE231350. Source data and source code can be found in S1 Data and S1 Code.
(PNG)

S2 Fig Transcriptomics and protein expression upon vehicle or docetaxel treatment. (A) Heatmap showing the top 10 DEGs in each cluster in a merged dataset. (B) Volcano plot showing significantly differentially expressed protein-coding genes in single-cell suspension of tumor stromal organoids based on scRNA-seq data from docetaxel treated compared with vehicle-treated controls. Transcripts with FC > 0.5 and adjusted *P*-value < 0.05 are highlighted in red. (C) Dot plots of Il6 and Csf3 genes from merged samples. Dot size indicates the proportion of cells in each cluster expressing a gene and color shading indicates the relative level of gene expression. (D) Violin plots showing Il6 and Csf3 gene expression levels in D2.0R 2 cancer cell cluster (top) and Fibro/CAF1stromal cell cluster (bottom). (E) Representative western blot image showing MEK, p-ERK, ERK, and GAPDH (loading control) and quantification of band intensity relative to loading control (*n* = 3). Independent *t* test measurement of band intensities of proteins shows statistical significance between docetaxel and vehicle treatment. Raw blot images can be found in S1 Raw Image. (F) EdU incorporation assay showing EdU staining (green), Hoechst 33342 (blue), and D2.0R cells (red) in tumor stromal organoids cultured in RGF-BME; scale bar = 330 μm. scRNA-seq source data is available on GEO (accession # GSE231350). Source data and source code can be found in S1 Data and S1 Code.
(PNG)

S3 Fig Awakened cancer cells did not succumb to repeated cycles of chemotherapy. (A) Representative BLI flux kinetics of D2.0R luc-mCherry tumor growth in the mfp of untreated mice followed for approximately 4 months (*n* = 6). (B, C) Representative BLI flux kinetics (B) and bioluminescence images (C) of D2.0R luc-mCherry tumor growth in the mfp of mice treated with 3 cycles of docetaxel (*n* = 5). Source data can be found in S1 Data.
(PNG)

S4 Fig Tumor immunosuppressive signature upon docetaxel treatment. (A, B) Ratio of M1:M2 macrophages (A) and CD8:Tregs (B) in the mfps/tumors of mice treated with vehicle or docetaxel (*n* = 3, each). Independent *t* test measurement shows statistical significance between treatment groups. Source data can be found in S1 Data.
(PNG)

S5 Fig Transcriptomics analysis-cluster markers, cell–cell communication, and cancer cell phenotypes. (A) Cell type identifying cluster markers with clusters grouped by major phenotype classifying genes. (B) UMAP presentation of major immune cell types and associated clusters in vehicle (VEH) and docetaxel (DTX) treated murine mfp/tumor (*n* = 2, each treatment group). (C) Heatmap of differential interactions between the different cell types in VEH and DTX treated groups: Rows and columns represent source and target clusters, respectively. Gradience in the color shows lowest (light) to highest (dark) number of networks; red gradient—Vehicle and blue gradient—DTX. Bar plots on the right and top of the heatmap represent the total outgoing and incoming interaction scores, respectively. (D) Chord connections between cell types shows Il17 signaling network in DTX dataset. (E) Chord connections between cell types shows Spp1 signaling network in VEH and DTX datasets, respectively. (F) Dot plot of Ki67 gene expression in cancer cells by treatment. (G) Differentially regulated ingenuity pathways enriched or down-regulated upon docetaxel treatment compared with vehicle controls in cancer cells in the mfp/tumor based on differentially expressed RNAs. -log (*p*-value) for each pathway are indicated on the x-axis. Color red or green indicates positive or negative z-score, respectively. (H) Dot plots of selected markers in cancer cells 1 and cancer cells 2 from merged dataset (top) and grouped by treatment, VEH and DTX (bottom). Dot size indicates the proportion of cells in each cluster expressing a gene and color shading indicates the relative level of gene expression. scRNA-seq source data is available on GEO (accession # GSE231350). Source data and source code can be found in S1 Data and S1 Code.
(PNG)

S6 Fig Cell cycle genes expression in stromal cells in mammary tumor of vehicle and docetaxel-treated mice. Dot plots of S phase (top) and G2M phase (bottom) cell cycle genes in stromal cells grouped by treatment, VEH and DTX. Dot size indicates the proportion of cells in each cluster expressing a gene and color shading indicates the relative level of gene expression. scRNA-seq source data is available on GEO (accession # GSE231350). Source code can be found in S1 Code.
(PNG)

S7 Fig Gene set analysis for evaluating clinical relevance. (A) Gene set enrichment plot of 10 gene signature in breast cancer. (B) Box plot compares the gene set variation analysis score between tumor and normal samples. (C, D) Kaplan–Meier curves for disease-specific survival (DSS) and disease-free interval (DFI) between high and low GSVA score groups in breast cancer. (E) Trend of GSVA score between stages in breast cancer. (F) Heatmap shows correlation between GSVA score and activity of cancer-related pathways in breast cancer. Supporting information can be found in S3–S6 Tables.
(PNG)

S8 Fig Flow gating strategy for cancer cells, myeloid cells, and T cells in vehicle and docetaxel-treated murine mammary fat pad/tumors. Raw flow cytometry data is deposited on Flow repository (FR-FCM-Z6J8).
(PNG)

S1 Table Dormancy associated genes up-regulated in cancer cells in TSO relative to D2.0R 3D. (PNG)

S2 Table 66-genes enriched upon chemotherapy in both in vitro and in vivo datasets. (PNG)

S3 Table Table presents the survival difference between high and low gene expression groups. (XLSX)

S4 Table GSVA analysis of the gene set to evaluate survival probability. (XLSX)

S5 Table GSVA analyses shows changes in gene set relevant to pathologic stages and cancer subtypes. (XLSX)

S6 Table Analysis parameters for RFS probability with the 10-gene signature on KM-plotter. (XLSX)

S1 Data Source data. (XLSX)

S1 Code Source code. (RTF)

S1 Raw Image Raw blot image for S2E Fig. (PDF)

S2 Raw Image Raw blot image for Fig 3H. (PDF)

## Abbreviation

CAF: cancer associated fibroblast
CM: conditioned media
DTX: docetaxel
FUCCI: fluorescence ubiquitination cell cycle indicator
G-CSF: granulocyte colony stimulating factor
HE: hematoxylin and eosin
IACUC: Institutional Animal Care and Use Committee
IL-6: interleukin-6
IPA: ingenuity pathways analysis
MEF: murine embryonic fibroblast
mfp: mammary fat pad
NES: normalized enrichment score
PCA: principal component analysis
scRNA-seq: single-cell RNA sequencing
TME: tumor microenvironment
TSO: tumor stromal organoid
TSS: tumor stromal spheroid
UMAP: Uniform Manifold Approximation and Projection
VEH: vehicle
