# Supplementary figures and images for: Taxane chemotherapy induces stromal injury that leads to breast cancer dormancy escape
Source: PLoS Biol. 2023 Sep 12;21(9):e3002275. doi: 10.1371/journal.pbio.3002275 (PMC10497165; doi:10.1371/journal.pbio.3002275)

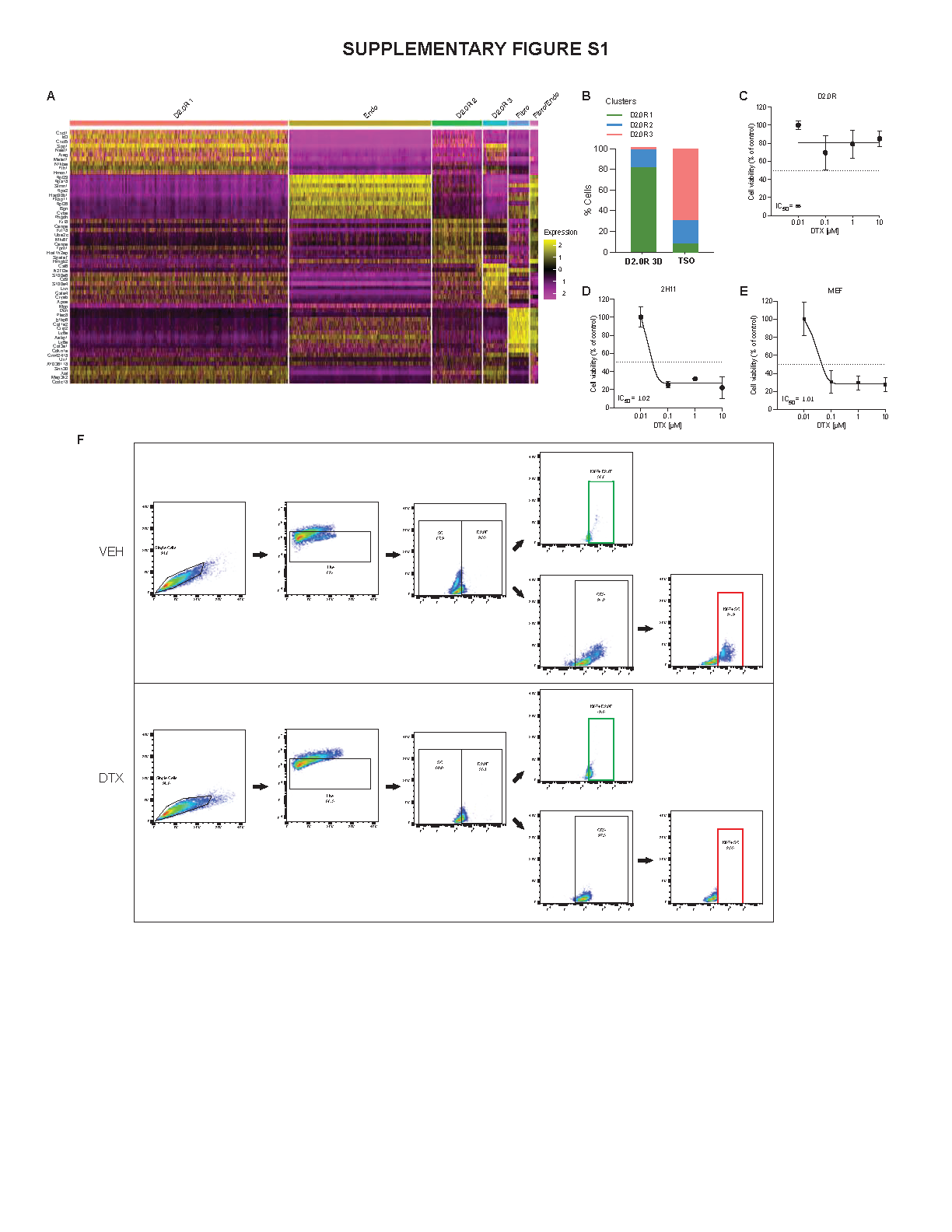

Supplement: S1 Fig — (A) Heatmap showing the top 10 DEGs in each cluster in a merged dataset. (B) Percentage of the different clusters of cancer cells from D2.0R 3D and TSO, per total cancer cells in the respective datasets. (C–E) Dose response curves of cancer cells (D2.0R) (n = 5), endothelial (2H11) (n = 4), and fibroblasts (MEF) (n = 3) to varying concentrations of DTX (0–10 μm). (F) Representative images of flow gating strategy for singlets, live cells, and Ki67+ D2.0Rs (mCherry) (green box) or Ki-67+ stromal cells (2H11:MEF) (red box) in VEH and DTX treated TSOs. scRNA-seq files are accessible on GEO with the accession number GSE231350. Source data and source code can be found in S1 Data and S1 Code. (PNG) [file pbio.3002275.s001.png]

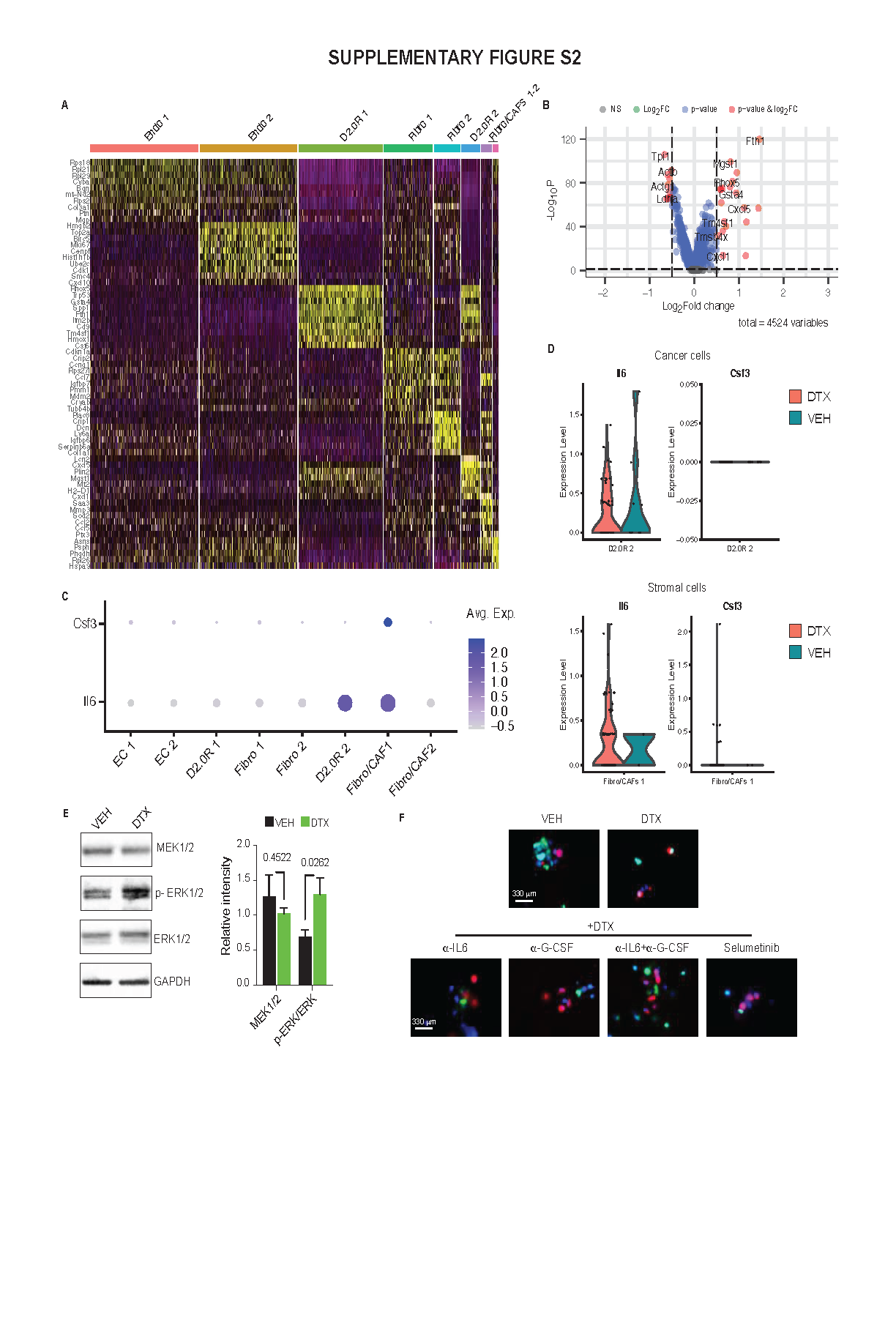

Supplement: S2 Fig — (A) Heatmap showing the top 10 DEGs in each cluster in a merged dataset. (B) Volcano plot showing significantly differentially expressed protein-coding genes in single-cell suspension of tumor stromal organoids based on scRNA-seq data from docetaxel treated compared with vehicle-treated controls. Transcripts with FC > 0.5 and adjusted P-value < 0.05 are highlighted in red. (C) Dot plots of Il6 and Csf3 genes from merged samples. Dot size indicates the proportion of cells in each cluster expressing a gene and color shading indicates the relative level of gene expression. (D) Violin plots showing Il6 and Csf3 gene expression levels in D2.0R 2 cancer cell cluster (top) and Fibro/CAF1stromal cell cluster (bottom). (E) Representative western blot image showing MEK, p-ERK, ERK, and GAPDH (loading control) and quantification of band intensity relative to loading control (n = 3). Independent t test measurement of band intensities of proteins shows statistical significance between docetaxel and vehicle treatment. Raw blot images can be found in S1 Raw Image. (F) EdU incorporation assay showing EdU staining (green), Hoechst 33342 (blue), and D2.0R cells (red) in tumor stromal organoids cultured in RGF-BME; scale bar = 330 μm. scRNA-seq source data is available on GEO (accession # GSE231350). Source data and source code can be found in S1 Data and S1 Code. (PNG) [file pbio.3002275.s002.png]

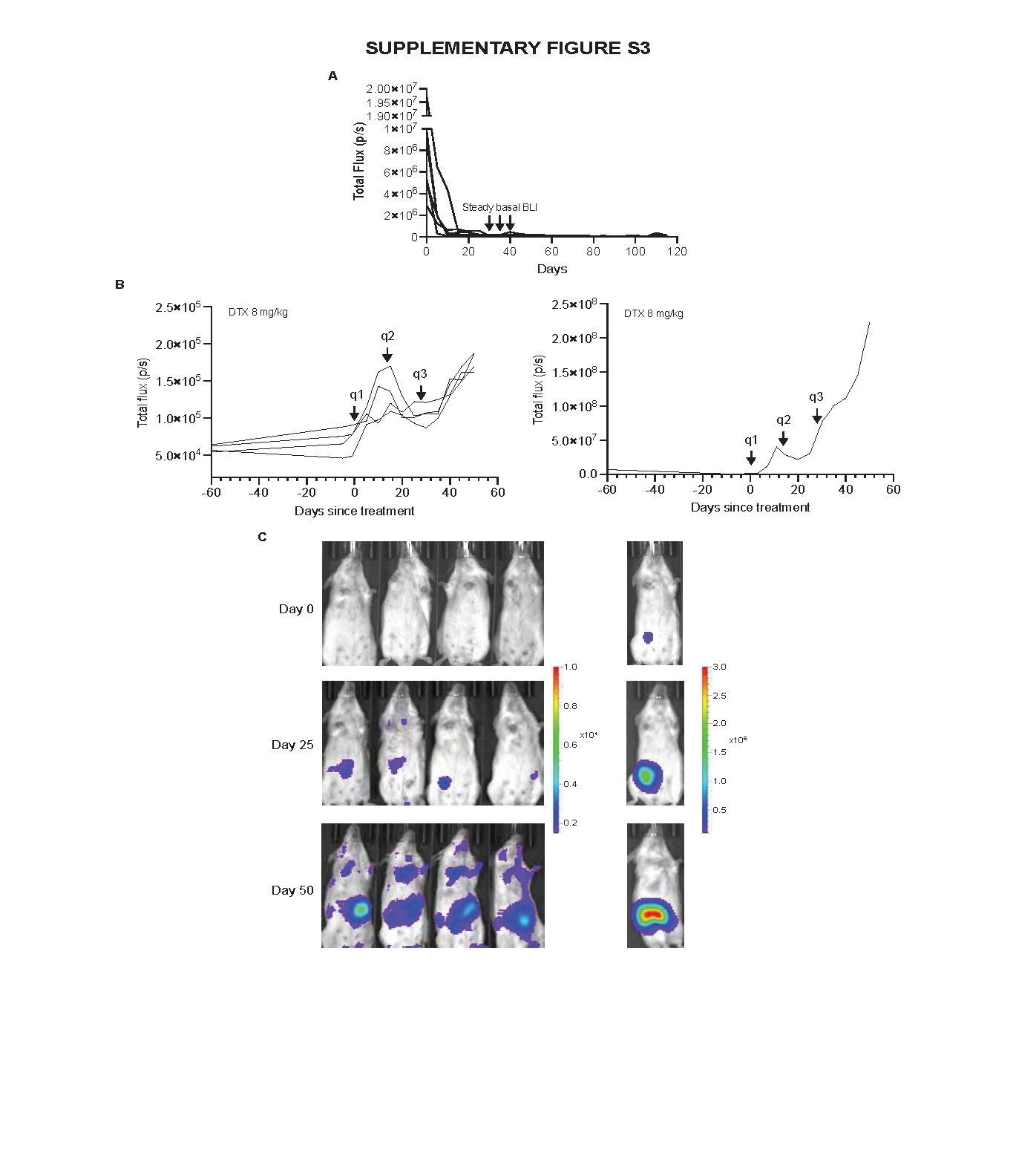

Supplement: S3 Fig — (A) Representative BLI flux kinetics of D2.0R luc-mCherry tumor growth in the mfp of untreated mice followed for approximately 4 months (n = 6). (B, C) Representative BLI flux kinetics (B) and bioluminescence images (C) of D2.0R luc-mCherry tumor growth in the mfp of mice treated with 3 cycles of docetaxel (n = 5). Source data can be found in S1 Data. (PNG) [file pbio.3002275.s003.png]

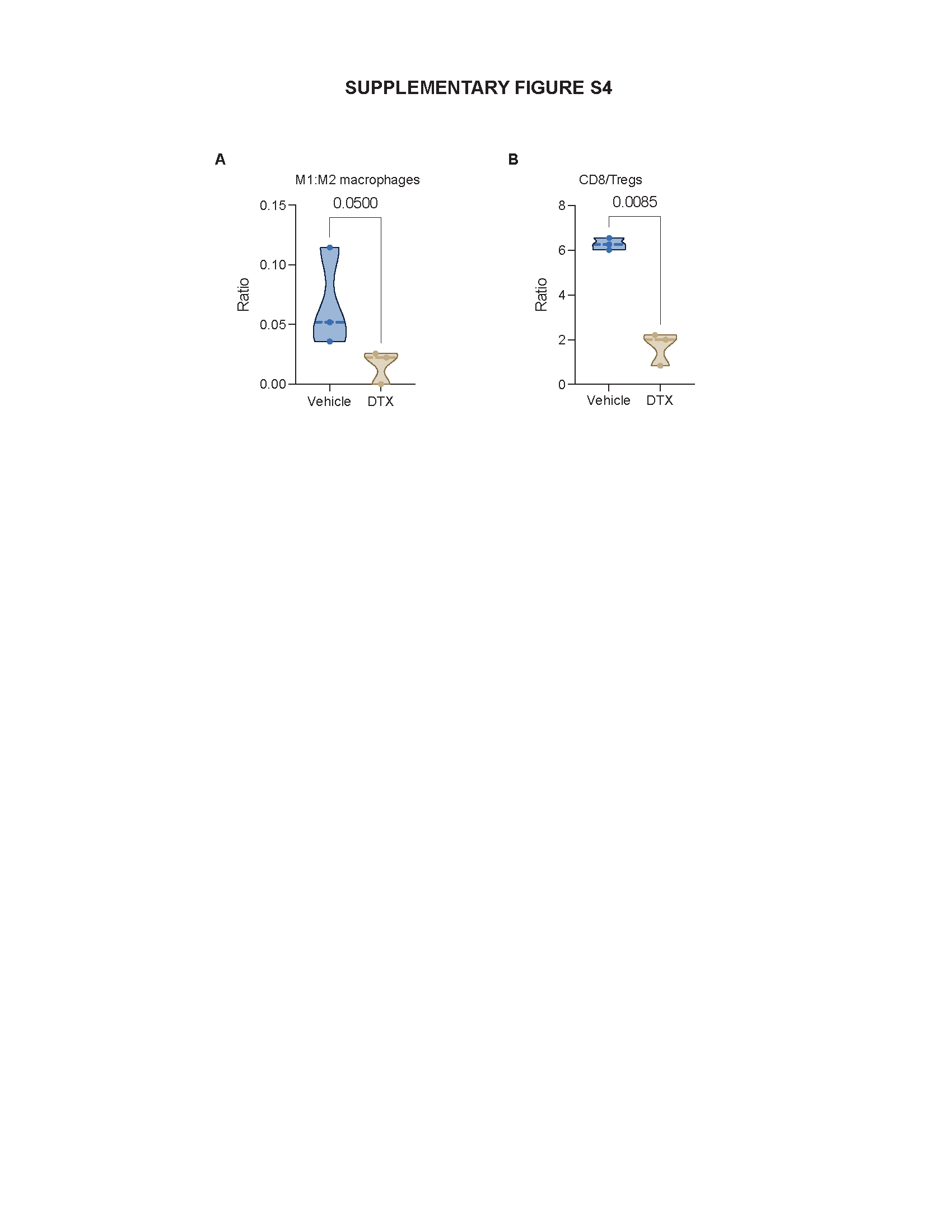

Supplement: S4 Fig — (A, B) Ratio of M1:M2 macrophages (A) and CD8:Tregs (B) in the mfps/tumors of mice treated with vehicle or docetaxel (n = 3, each). Independent t test measurement shows statistical significance between treatment groups. Source data can be found in S1 Data. (PNG) [file pbio.3002275.s004.png]

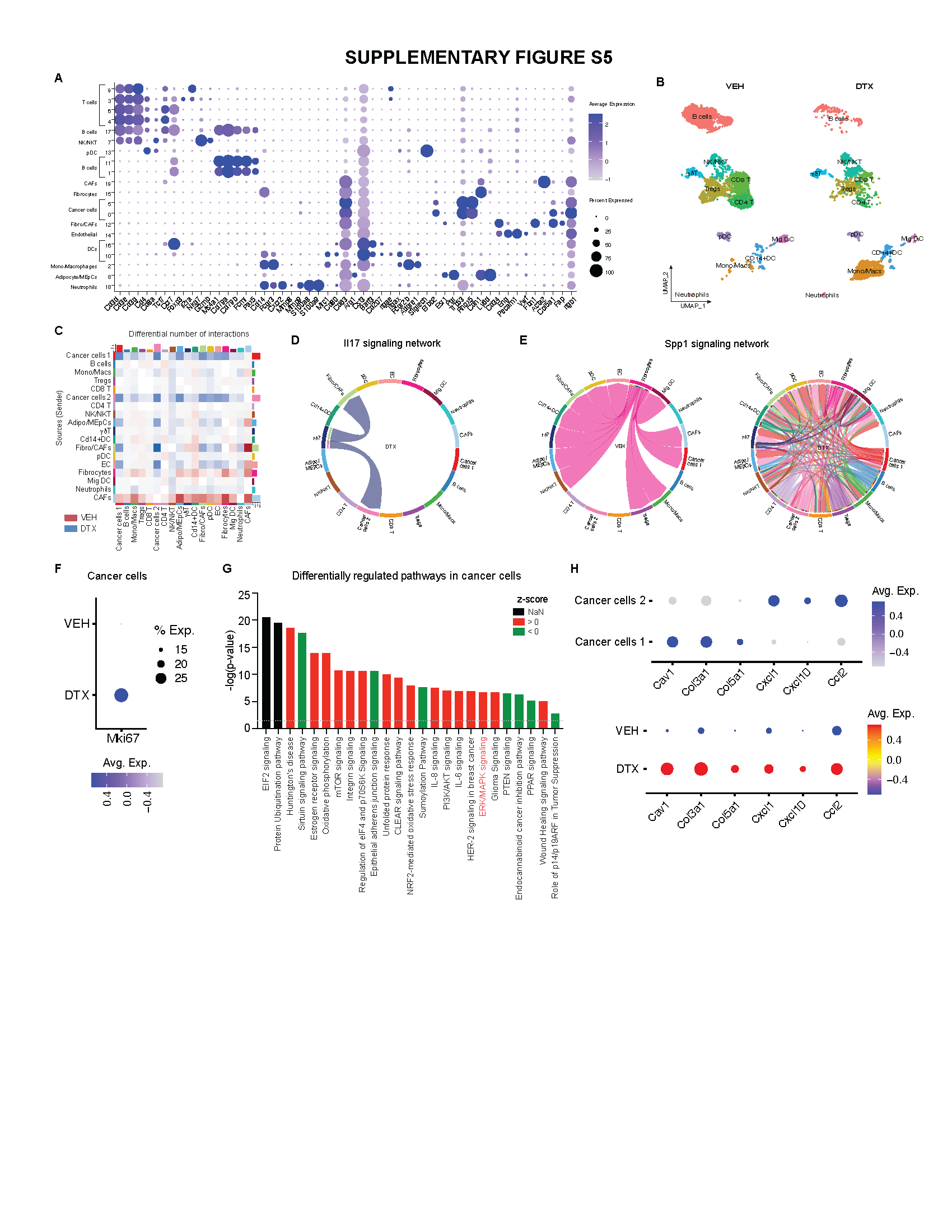

Supplement: S5 Fig — (A) Cell type identifying cluster markers with clusters grouped by major phenotype classifying genes. (B) UMAP presentation of major immune cell types and associated clusters in vehicle (VEH) and docetaxel (DTX) treated murine mfp/tumor (n = 2, each treatment group). (C) Heatmap of differential interactions between the different cell types in VEH and DTX treated groups: Rows and columns represent source and target clusters, respectively. Gradience in the color shows lowest (light) to highest (dark) number of networks; red gradient—Vehicle and blue gradient—DTX. Bar plots on the right and top of the heatmap represent the total outgoing and incoming interaction scores, respectively. (D) Chord connections between cell types shows Il17 signaling network in DTX dataset. (E) Chord connections between cell types shows Spp1 signaling network in VEH and DTX datasets, respectively. (F) Dot plot of Ki67 gene expression in cancer cells by treatment. (G) Differentially regulated ingenuity pathways enriched or down-regulated upon docetaxel treatment compared with vehicle controls in cancer cells in the mfp/tumor based on differentially expressed RNAs. -log (p-value) for each pathway are indicated on the x-axis. Color red or green indicates positive or negative z-score, respectively. (H) Dot plots of selected markers in cancer cells 1 and cancer cells 2 from merged dataset (top) and grouped by treatment, VEH and DTX (bottom). Dot size indicates the proportion of cells in each cluster expressing a gene and color shading indicates the relative level of gene expression. scRNA-seq source data is available on GEO (accession # GSE231350). Source data and source code can be found in S1 Data and S1 Code. (PNG) [file pbio.3002275.s005.png]

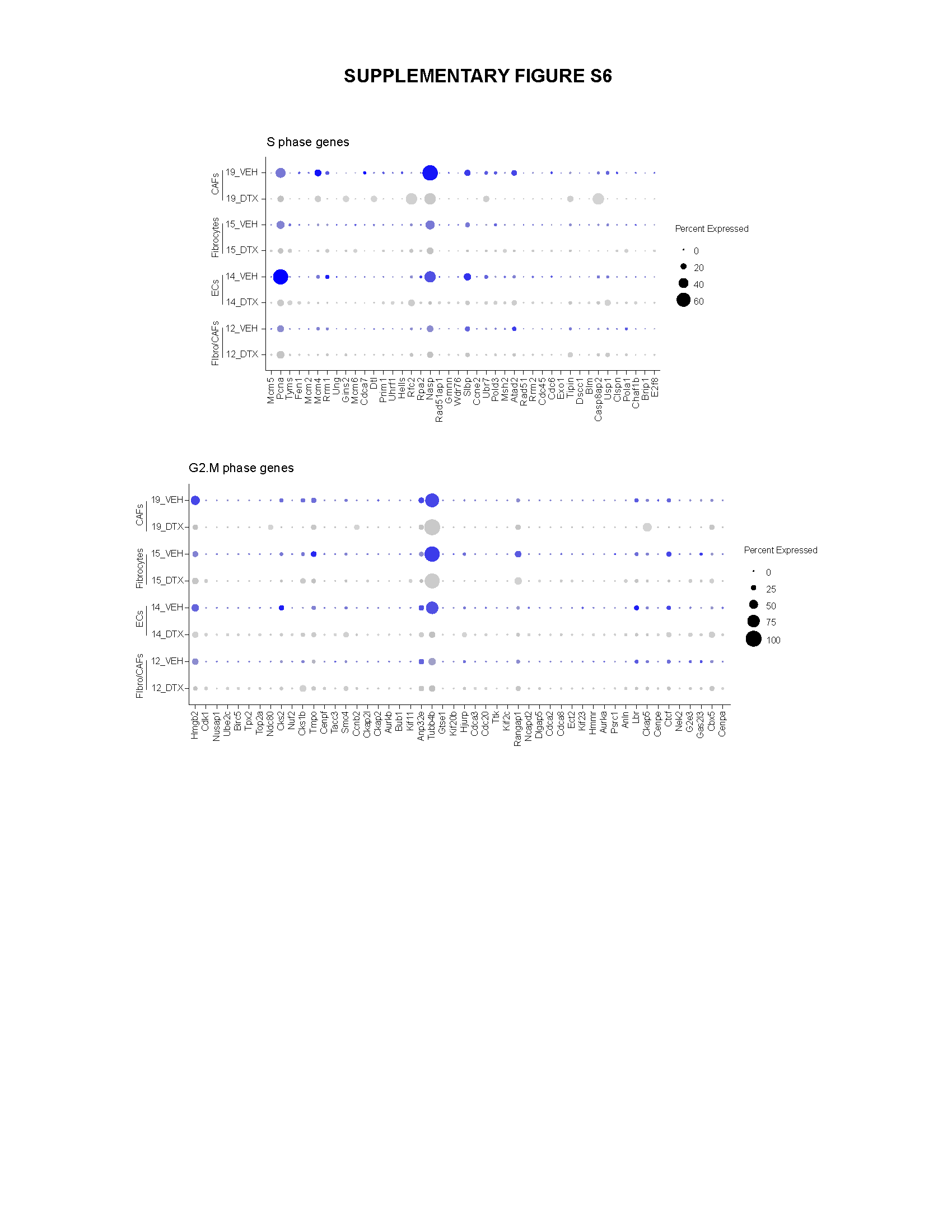

Supplement: S6 Fig — Dot plots of S phase (top) and G2M phase (bottom) cell cycle genes in stromal cells grouped by treatment, VEH and DTX. Dot size indicates the proportion of cells in each cluster expressing a gene and color shading indicates the relative level of gene expression. scRNA-seq source data is available on GEO (accession # GSE231350). Source code can be found in S1 Code. (PNG) [file pbio.3002275.s006.png]

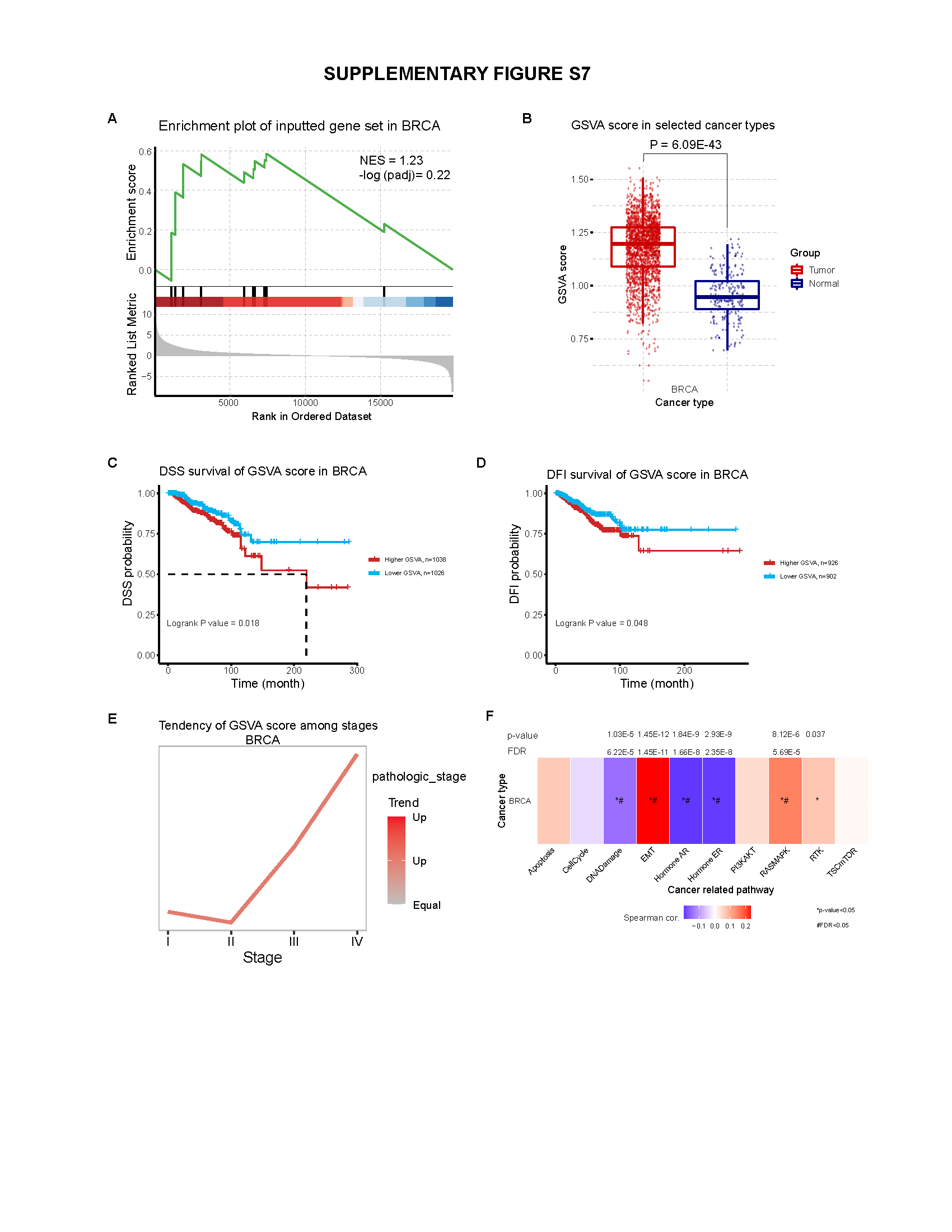

Supplement: S7 Fig — (A) Gene set enrichment plot of 10 gene signature in breast cancer. (B) Box plot compares the gene set variation analysis score between tumor and normal samples. (C, D) Kaplan–Meier curves for disease-specific survival (DSS) and disease-free interval (DFI) between high and low GSVA score groups in breast cancer. (E) Trend of GSVA score between stages in breast cancer. (F) Heatmap shows correlation between GSVA score and activity of cancer-related pathways in breast cancer. Supporting information can be found in S3–S6 Tables. (PNG) [file pbio.3002275.s007.png]

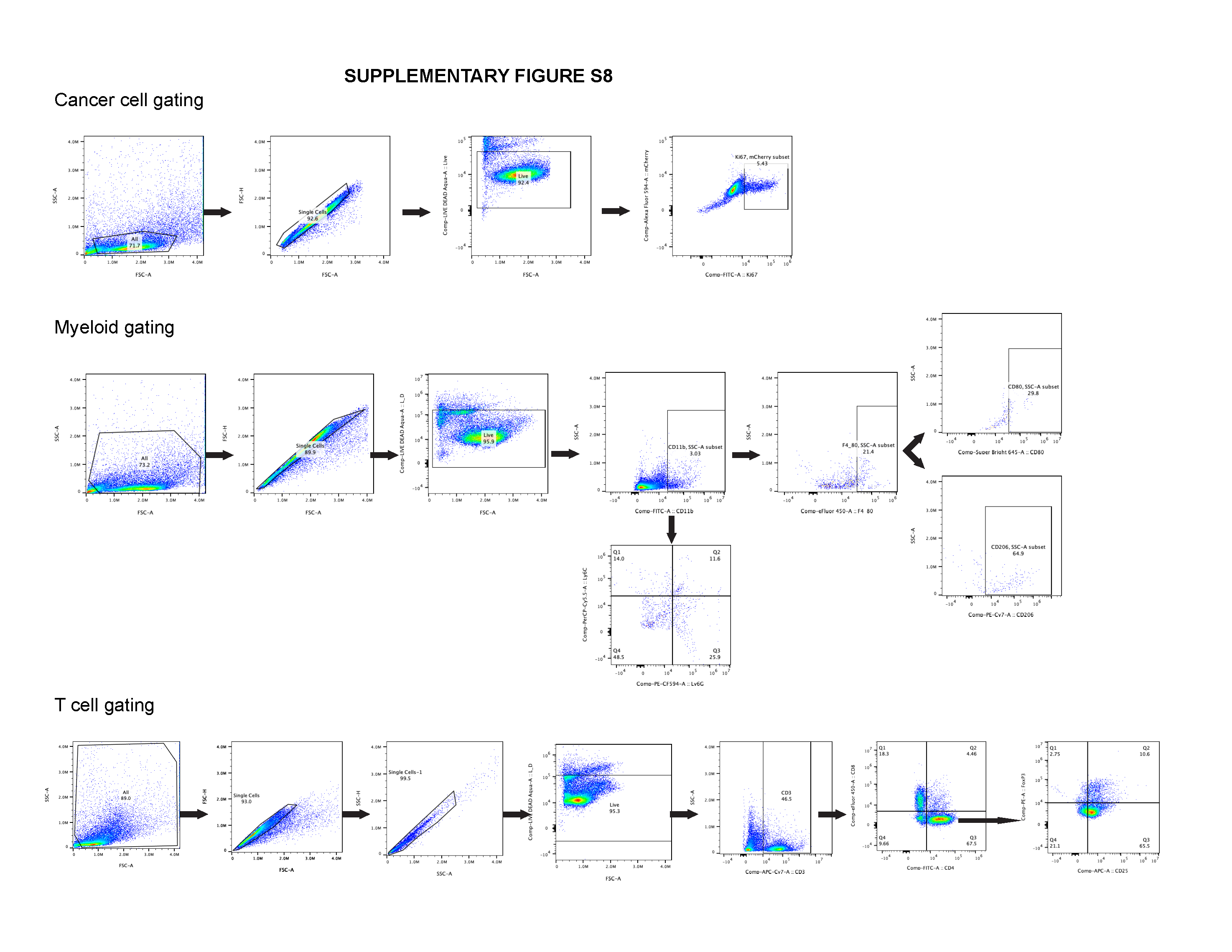

Supplement: S8 Fig — Raw flow cytometry data is deposited on Flow repository (FR-FCM-Z6J8). (PNG) [file pbio.3002275.s008.png]

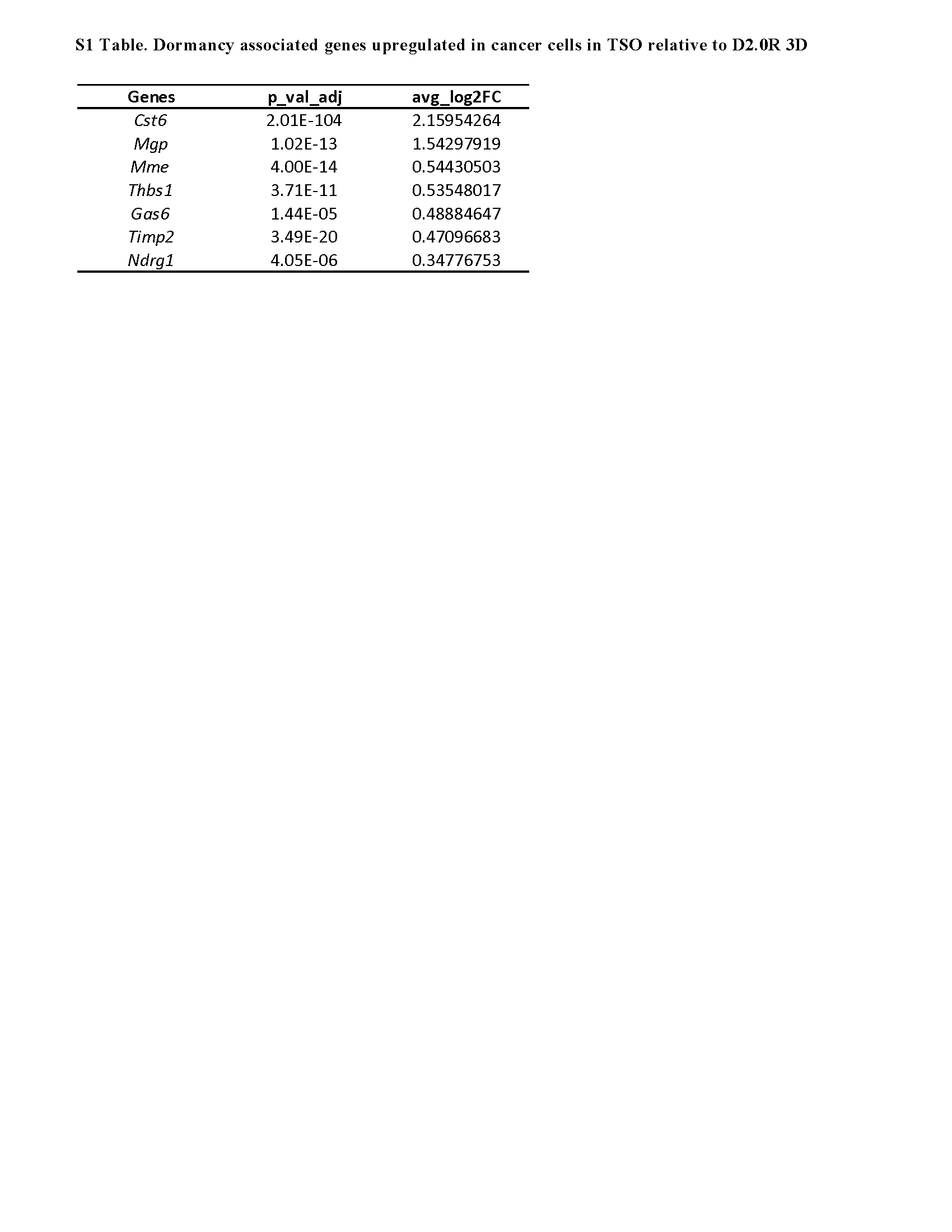

Supplement: S1 Table — (PNG) [file pbio.3002275.s009.png]

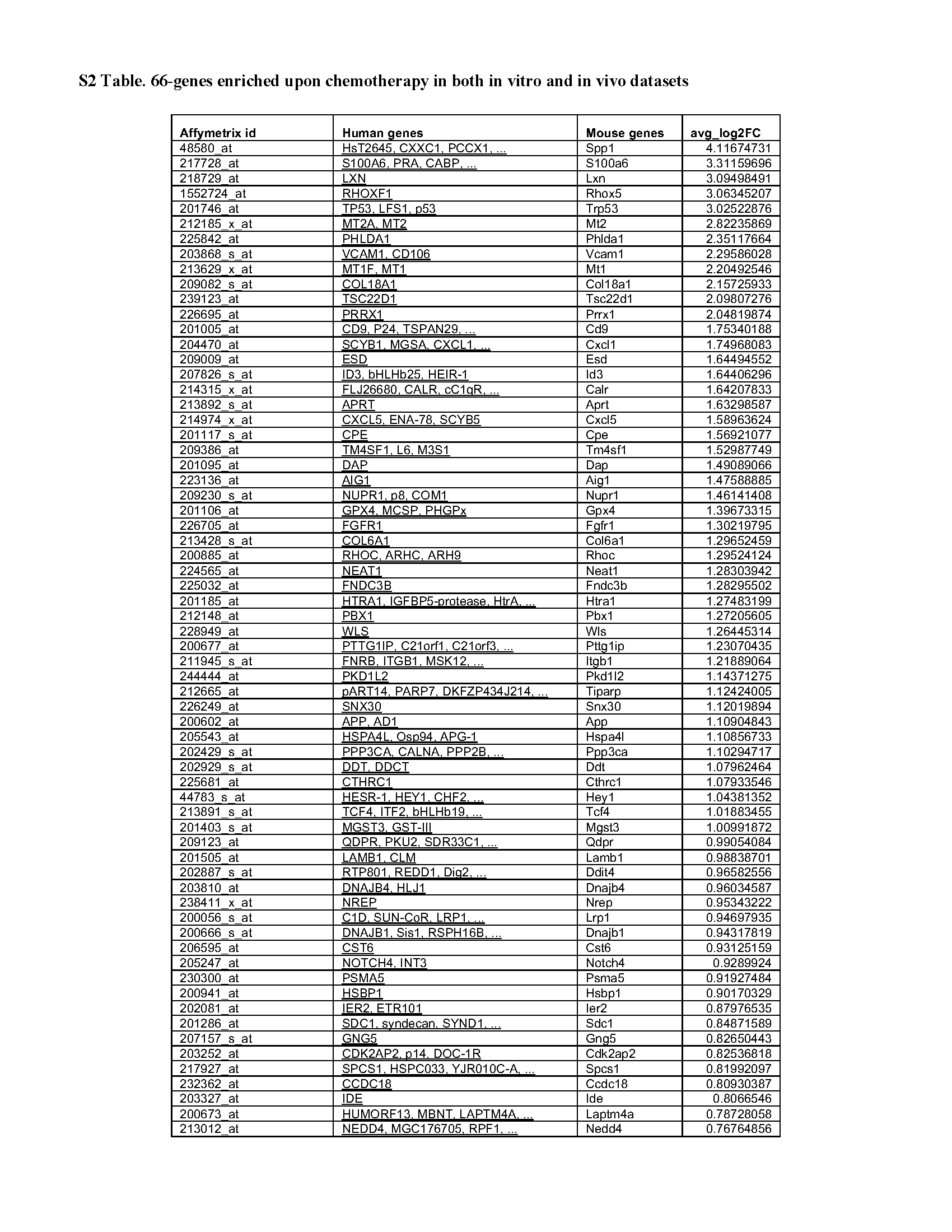

Supplement: S2 Table — (PNG) [file pbio.3002275.s010.png]

Raw blot images for supplementary figure S2E

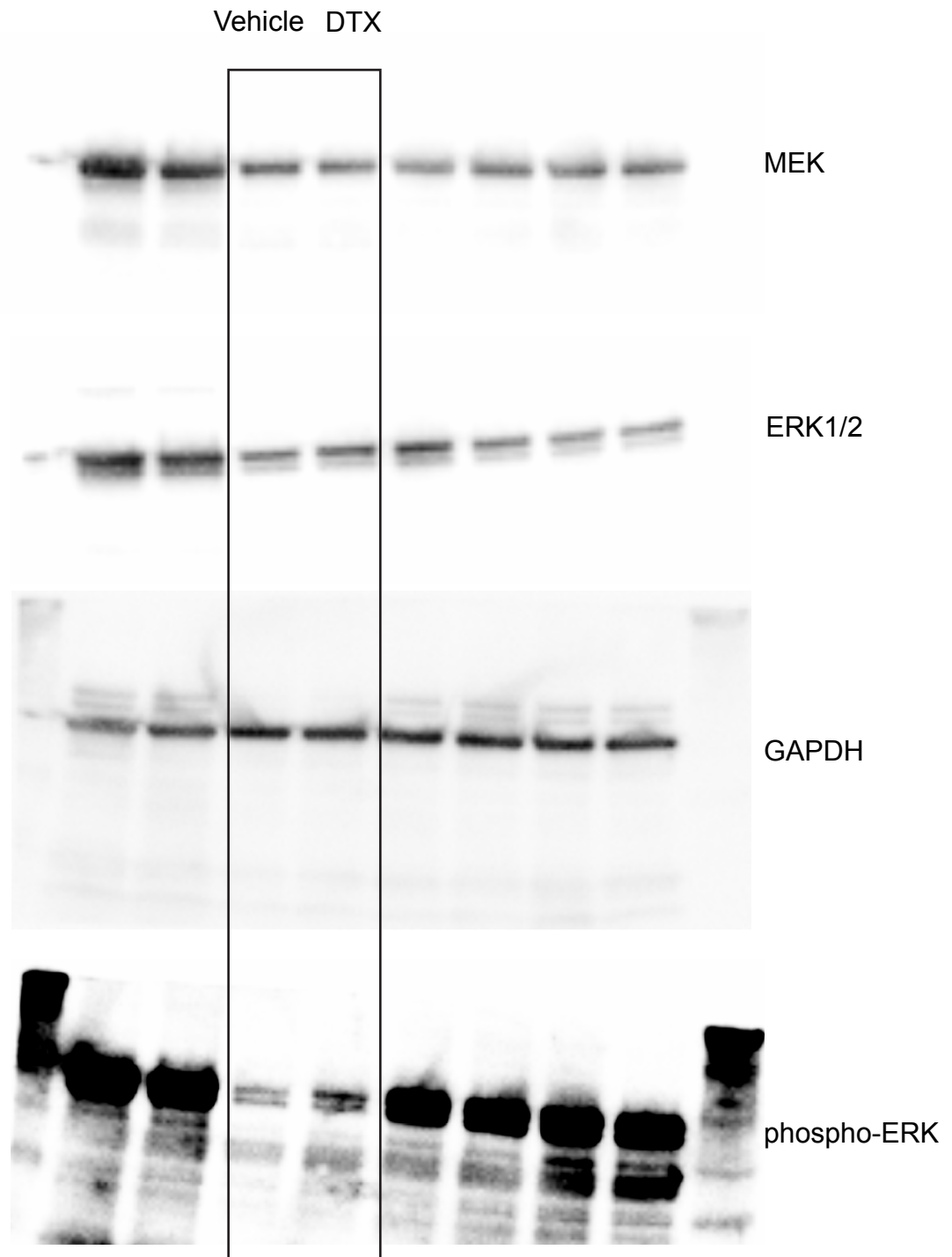

Supplement: S1 Raw Image — (PDF) [file pbio.3002275.s017.pdf]

Raw blot images - Figure 3H

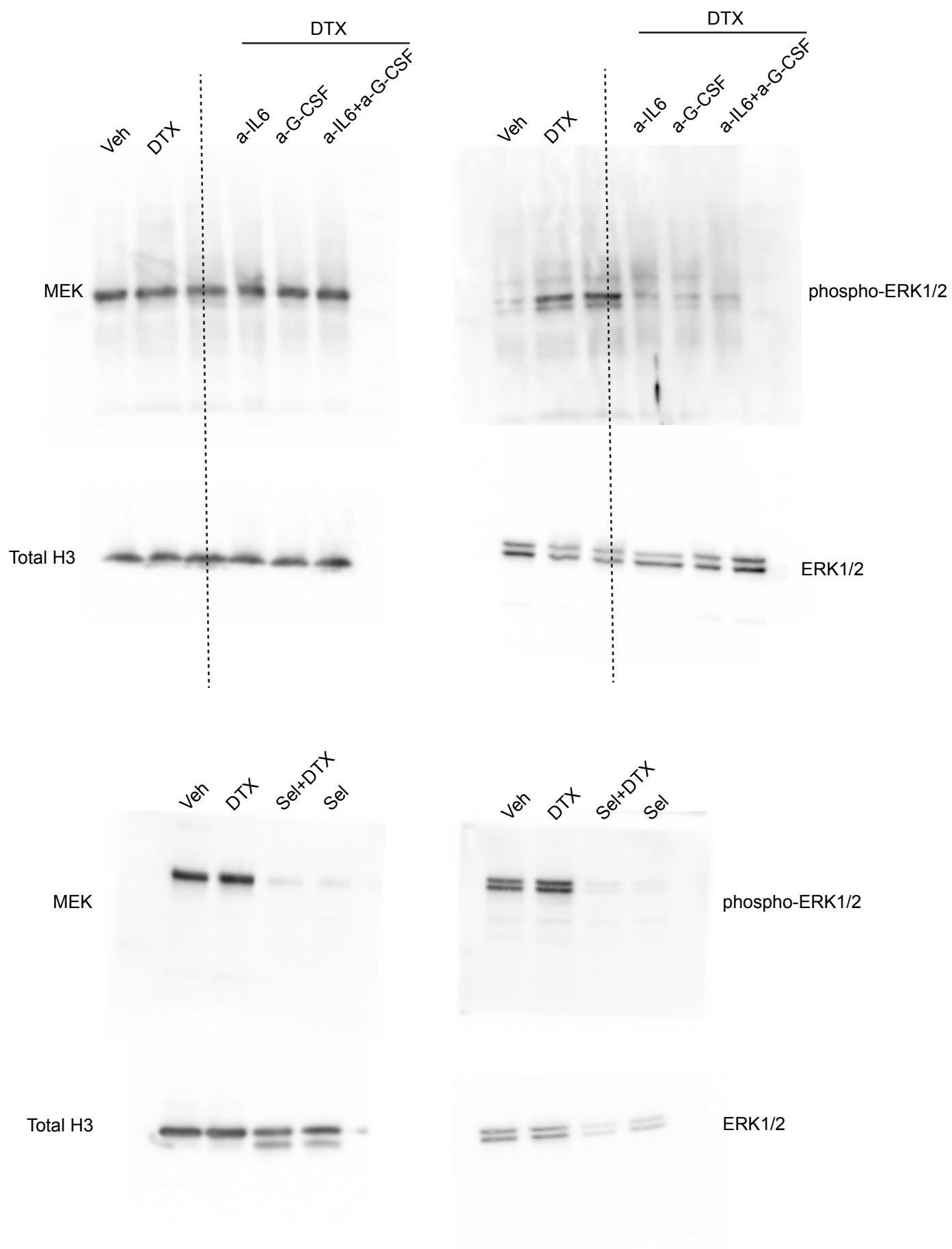

Supplement: S2 Raw Image — (PDF) [file pbio.3002275.s018.pdf]
