# Supplementary material for: Taxane chemotherapy induces stromal injury that leads to breast cancer dormancy escape
Source: PLoS Biol. 2023 Sep 12;21(9):e3002275. doi: 10.1371/journal.pbio.3002275 (PMC10497165; doi:10.1371/journal.pbio.3002275)
Supplement: S1 Code — (RTF) [file pbio.3002275.s016.rtf]

Source code for figures 1, S1, 3, S2, 5, 6, S5 and S6

library(RColorBrewer)
library(ggplot2)
library(plyr)
library(Seurat)
library(dplyr)
library(Matrix)
library(data.table)
library(magrittr)

Figure 1 and Supplementary Fig S1

##Rename groups
>tcc3d$group <- revalue(tcc3d_final$group, c("3D cells" = "D2.OR 3D", "Trip. co-culture" = "TSO"))

##UMAP by group (Fig 1A)
> DimPlot(tcc3d_final, reduction = "umap", label = TRUE, pt.size = 0.5, label.size = 3, group.by = "group")

##UMAP of merged dataset (Fig 1B)
> DimPlot(tcc3d_final, reduction = "umap", label = TRUE, pt.size = 0.5, label.size = 3)

##Cluster Markers heat map (Fig S1A)
> tcc3d.markers <- FindAllMarkers(object = tcc3d_final, only.pos = TRUE, min.pct = 0.25, logfc.threshold = 0.25)
> tcc3d.markers = tcc3d.markers[!grepl("mt-", tcc3d.markers$gene),]
> top10 <- tcc3d.markers %>% group_by(cluster) %>% top_n(n = 10, wt = avg_log2FC)
> DoHeatmap(tcc3d_final, features = top10$gene, size = 2.5)

##Subset Cancer cell clusters
> ClusD20R<-subset(tcc3d, idents = c("D2.0R 1", "D2.0R 2", "D2.0R 3"))

##Cluster Markers heat map in cancer cells subset (Fig 1C)
> D20R<-FindAllMarkers(ClusD20R, only.pos = TRUE, min.pct = 0.25, logfc.threshold = 0.25)
>  top10 <- D20R %>% group_by(cluster) %>% top_n(n = 10, wt = avg_log2FC)
> DoHeatmap(ClusD20R, features = top10$gene, size = 2.5)

##Getting cell numbers by cluster (Fig S1B)
> md <- ClusD20R@meta.data %>% as.data.table
> md[, .N, by = c("sample", "seurat_clusters")] %>% dcast(., sample ~ seurat_clusters, value.var = "N")

##Finding DEGs in cancer cell clusters in TSO vs D2.0R 3D (Table S1)
> Idents(ClusD20R) <- ClusD20R$group
> D20RTCCv3D<-FindMarkers(ClusD20R, ident.1 = "TSO", ident.2 = "D2.OR 3D", only.pos = TRUE, min.pct = 0.25, logfc.threshold = 0.25) 
> write.table(D20RTCCv3D,file = "D20RTCCv3D.csv",sep=',')


Figure 3 and Supplementary Fig S2

##Rename groups
>s2s3$group <- revalue(s2s3$group, c(“Veh” = “VEH”, “DTX” = “DTX”))

##UMAP of merged dataset (Fig 3A)
> DimPlot(s2s3, reduction = "umap", label = TRUE, pt.size = 0.5, label.size = 3, split.by = "group")

##Cluster Markers heat map (Fig S2A)
##Rename clusters
> new.cluster.ids <- c(“Endo 1", "Endo 2", "D2.0R 1", "Fibro 1", "Fibro 2", "D2.0R 2", "Fibro/CAF 1", "Fibro/CAF 2")
> names(new.cluster.ids) <- levels(s2s3)
> s2s3 <- RenameIdents(s2s3, new.cluster.ids)


> s2s3.markers <- FindAllMarkers(object = s2s3, only.pos = TRUE, min.pct = 0.25, logfc.threshold = 0.25)
> s2s3.markers = s2s3.markers[!grepl("mt-", s2s3.markers$gene),]
> top10 <- s2s3.markers %>% group_by(cluster) %>% top_n(n = 10, wt = avg_log2FC)
> DoHeatmap(s2s3, features = top10$gene, size = 2.5)

##DEGs in DTX v VEH (Fig 3B and Fig S2B)
> Idents(s2s3) <- s2s3$group
> s2s3DTXvVEH<-FindMarkers(s2s3, ident.1 = "DTX", ident.2 = "VEH", only.pos = TRUE, min.pct = 0.25, logfc.threshold = 0.25)
>  top10 <- s2s3DTXvVEH %>% group_by(group) %>% top_n(n = 10, wt = avg_log2FC)
>  DoHeatmap(s2s3, features = top10$gene, size = 2.5)
>EnhancedVolcano(s2s3DTXvVEH, lab = rownames(s2s3DTXvVEH), x = 'avg_log2FC',y = 'p_val', pCutoff = 0.05, FCcutoff = 0.5, pointSize = 1.0, labSize = 3)

##Subset Cancer cell clusters
> Idents(s2s3) <- s2s3$seurat_clusters
> ClusD2.0R<-subset(s2s3, idents = c("D2.0R 1", "D2.0R 2"))

##DEGs in cancer cell clusters DTX v VEH (Fig 3D)
> Idents(ClusD2.0R) <- ClusD2.0R$group
> D2.0RDTXvVEH<-FindMarkers(ClusD2.0R, ident.1 = "DTX", ident.2 = "VEH", only.pos = TRUE, min.pct = 0.25, logfc.threshold = 0.25)
>EnhancedVolcano(D2.0RDTXvVEH, lab = rownames(D2.0RDTXvVEH), x = 'avg_log2FC',y = 'p_val', pCutoff = 0.05, FCcutoff = 0.5, pointSize = 1.0, labSize = 3)

##Il6 and Csf3 gene expression in clusters (Fig S2C-D)
>DotPlot(s2s3, features = c('Il6', 'Csf3'))
> Idents(s2s3) <- s2s3$group
> VlnPlot(s2s3, features = c('Il6', 'Csf3'), idents =  c('D2.0R 2'), split.by =   "group")
> VlnPlot(s2s3, features = c('Il6', 'Csf3'), idents =  c('Fibro/CAF 1'), split.by =   "group")


Figures 5, 6 and Supplementary Figure S5

##Rename clusters
new.cluster.ids <- c("Cancer cells 1", "B cells", "Mono/Macs", "Tregs", "CD8 T", "Cancer cells 2", "CD4 T", "NK/NKT", "Adipo/MEpCs", "ãäT","cDC","B cells"," Fibro/CAFs", "pDC", "EC"," Fibrocytes", "Mig DC", "B cells”, "Neutrophils", "CAFs")
names(new.cluster.ids) <- levels(Veh_DTX)
Veh_DTX <- RenameIdents(Veh_DTX, new.cluster.ids)

##Subset immune-cells (Fig 5L)
> Immunecells<- subset(Veh_DTX, idents = c(“B cells", "Mono/Macs", "Tregs", "CD8 T", "CD4 T", "NK/NKT", "ãäT","cDC", "pDC", "Mig DC", "Neutrophils")
> DimPlot(Immunecells, reduction = "umap”, label = TRUE, pt.size = 0.5, label.size = 2) + NoLegend()

##Split PCA plot of immune cell clusters in the in vivo dataset (Supplementary Fig S5B)
>DimPlot(Immunecells, reduction = "umap", label = TRUE, pt.size = 0.5, split.by = "group", label.size = 2) + NoLegend()

##Getting cell numbers by cluster (Fig 5M, Fig 6B)
> md <- Veh_DTX@meta.data %>% as.data.table
> md[, .N, by = c("sample", "seurat_clusters")] %>% dcast(., sample ~ seurat_clusters, value.var = "N")

##Pro-tumor cytokines and chemokine (Fig 5N)
> DotPlot(Veh_DTX, features = c('Ccl5','Ccl8', 'Cxcl2', 'Cxcl1', 'Mmp9','Vegfa', 'Tgfb1', 'Tnf', 'Fn1', 'Spp1', 'Hilpda', 'Hmox1', 'Pdcd1','Cd274', 'Lag3', 'Havcr2'), idents = c('B cells', 'Mono/Macs', 'Tregs', 'CD8 T', 'CD4 T', 'NK/NKT', 'ãäT','Cd14+DC', 'pDC', 'Mig DC', 'Neutrophils'))

> DotPlot(Veh_DTX, features = c('Ccl2', 'Ccl5','Ccl8', 'Cxcl2', 'Cxcl1', 'Mmp9','Vegfa', 'Tgfb1', 'Tnf', 'Fn1', 'Spp1', 'Hilpda', 'Hmox1', 'Pdcd1','Cd274', 'Lag3', 'Havcr2'), idents = c('B cells', 'Mono/Macs', 'Tregs', 'CD8 T', 'CD4 T', 'NK/NKT', 'ãäT','Cd14+DC', 'pDC', 'Mig DC', 'Neutrophils'), group.by = "group")

##PCA plot of the complete in vivo dataset (Fig 6A)
>DimPlot(Veh_DTX, reduction = "umap", label = TRUE, pt.size = 0.5, label.size = 2) + NoLegend()

##Dot plot of Il6 and Csf3 genes expression (Fig 6C)
> DotPlot(Veh_DTX, features = c('Il6', 'Csf3'))

##DEGs in DTX v VEH complete in vivo dataset (Fig 6G)
> Idents(Veh_DTX) <- Veh_DTX$group
> DTXvVEH<-FindMarkers(Veh_DTX, ident.1 = "DTX", ident.2 = "VEH", only.pos = TRUE, min.pct = 0.25, logfc.threshold = 0.25)
>  top20 <- DTXvVEH %>% group_by(group) %>% top_n(n = 20, wt = avg_log2FC)
>  DoHeatmap(Veh_DTX, features = top20$gene, size = 2.5)

##Map2k2 feature plot with same scale (Fig 6I)
FeaturePlot(Veh_DTX, features= c('Map2k2'), split.by = "group", keep.scale = "all") & theme(legend.position = "right")

##DotPlot for cluster annotation (Supplementary Fig S5A)
> DotPlot(Veh_DTX, features = c('Cd3d', 'Cd3e', 'Cd3g', 'Cd4', 'Cd8a', 'Tcf7', 'Ccr7', 'Foxp3', 'Il2ra', 'Nkg7', 'Gzmb', 'Ms4a1', 'Cd79a', 'Cd79b', 'Fcmr', 'Pax5', 'Cd14', 'Fcgr3', 'Cxcr2', 'Mmp8', 'Mmp9', 'S100a8', 'S100a9', 'Mrc1', 'Cd80', 'Cd63', 'Arg1', 'Cst3', 'Batf3', 'Cd207', 'Itgae', 'Itgax', 'Fcgr2b', 'Adgre1', 'Siglech', 'Erbb2', 'Esr1', 'Pgr', 'Trp53', 'Rhox5', 'Cdh1', 'Ly6d', 'Cd34', 'Eng', 'Pecam1', 'Vwf', 'Fbn1', 'Acta2', 'Col5a1', 'Fap', 'Itgb1'))

##Subset cancer cells 
> Cancercells<- subset(Veh_DTX, idents = c("Cancer cells 1", "Cancer cells 2”))

##DotPlot for Mki67 gene expression in cancer cells (Supplementary figure S5F)
> Idents(Cancercells) <- Cancercells$group
>DotPlot(Cancercells, features = 'Mki67', group.by=group)

##DotPlot for CSC, invasiveness and inflammatory genes (Supplementary figure S5H)
> DotPlot(Cancercells, features = c('Cav1', 'Col3a1', 'Col5a1', 'Cxcl1', 'Cxcl10', 'Ccl2'))

>DotPlot(Cancercells, features = c('Cav1', 'Col3a1', 'Col5a1', 'Cxcl1', 'Cxcl10', 'Ccl2'), group.by = group)


## CellChat analysis (Figures 6D-F, and Supplementary figures S5C-E)

vd <- readRDS("Veh_Dtx.rds")

vd$cellTypes_chat <- vd$ann

dtx <- subset(vd, subset = group == "DTX")
dtx@active.assay <- "RNA"
dtx <- NormalizeData(dtx)
cellchat.dtx <- createCellChat(object = dtx, group.by = "cellTypes_chat")

veh <- subset(vd, subset = group == "VEH")
veh@active.assay <- "RNA"
veh <- NormalizeData(veh)
cellchat.veh <- createCellChat(object = veh, group.by = "cellTypes_chat")

object.list <- list(dtx = cellchat.dtx, veh = cellchat.veh)

CellChatDB <- CellChatDB.mouse
CellChatDB.use <- CellChatDB

for (i in 1:length(object.list)) {
o <- object.list[[i]]
o@DB <- CellChatDB.use

o <- subsetData(o)
o <- identifyOverExpressedGenes(o)
o <- identifyOverExpressedInteractions(o)
o <- projectData(o, PPI.mouse)

o <- computeCommunProb(o, raw.use = TRUE)
o <- filterCommunication(o, min.cells = 10)

o <- computeCommunProbPathway(o)

o <- aggregateNet(o)
object.list[[i]] <- o
print(paste(i,"Completed"))
}


cellchat <- mergeCellChat(object.list, add.names = names(object.list), cell.prefix = TRUE)

## Save CellChat Object ##
saveRDS(cellchat, file = "vd_cellchat_multi.rds")

## Identify signaling groups based on their functional similarity ##
cellchat <- computeNetSimilarityPairwise(cellchat, type = "functional", comparison = c(1,2))
cellchat <- netEmbedding(cellchat, type = "functional", comparison = c(1,2))
cellchat <- netClustering(cellchat, type = "functional", comparison = c(1,2))


pdf(file = "Merged4/CellChat/Figures/merge4_total_interactions.pdf")
gg1 <- compareInteractions(cellchat, show.legend = F, group = c(1,2))
gg2 <- compareInteractions(cellchat, show.legend = F, group = c(1,2), measure = "weight")
gg1 + gg2
dev.off()


Supplementary figure S5C
## Differential Number of Interactions and Interaction Strength ##
pdf(file = "Merged4/CellChat/Figures/merge4_diff_interactions_TB.pdf")
netVisual_diffInteraction(cellchat, weight.scale = T, comparison = c(1,2))
dev.off()


pdf(file = "Merged4/CellChat/Figures/merge4_diff_interactions_strength_TB.pdf")
netVisual_diffInteraction(cellchat, weight.scale = T, measure = "weight", comparison = c(1,2))
dev.off()


pdf(file = "Merged4/CellChat/Figures/merge4_diff_interactionsHeatmap_TB.pdf")
netVisual_heatmap(cellchat, comparison = c(1,2))
dev.off()


pdf(file = "Merged4/CellChat/Figures/merge4_diff_interactions_strengthHeatmap_TB.pdf")
netVisual_heatmap(cellchat, measure = "weight", comparison = c(1,2))
dev.off()


Figure 6F
## Compare the overall information flow of each signaling pathway ##
pdf(file = "Merged4/CellChat/Figures/information_flow.pdf")
gg1 <- rankNet(cellchat, mode = "comparison", stacked = T, do.stat = TRUE, comparison = c(1,2))
gg2 <- rankNet(cellchat, mode = "comparison", stacked = F, do.stat = TRUE, comparison = c(1,2))
gg1 + gg2
dev.off()


for (i in 1:length(object.list)) {
object.list[[i]] <- netAnalysis_computeCentrality(object.list[[i]])
}


Figure 6D-E, Supplementary figures S5D-E
## Signaling Chord Diagrams for each group in the significant pathways ##
pathways.show <- c("IL6", "CSF3", "SPP1", "Il17")
pdf("pathways_chord.pdf",sep = "", width = 12, height = 6)
for (i in 1:length(object.list)) {
netVisual_aggregate(object.list[[i]], signaling = pathways.show, layout = "chord", signaling.name = paste(pathways.show, names(object.list)[i]))
}
dev.off()


Supplementary Figure S6

## Dotplot of cell cycle associated genes expression in stromal cell in mammary tumor from vehicle and docetaxel treated mice

DTX_Veh<- readRDS
SCIV<-subset(DTX_Veh, idents = c('12', '14', '15', '19'))

sgenes <-c("Mcm5" , "Pcna" , "Tyms" , "Fen1" , "Mcm2" , "Mcm4"  ,"Rrm1" , "Ung" ,"Gins2" , "Mcm6" , "Cdca7", "Dtl"  , "Prim1" , "Uhrf1" , "Mlf1ip" , "Hells" , "Rfc2" , "Rpa2","Nasp" , "Rad51ap1", "Gmnn" , "Wdr76" , "Slbp" , "Ccne2" , "Ubr7" , "Pold3" , "Msh2" , "Atad2" , "Rad51" , "Rrm2" ,"Cdc45" , "Cdc6" , "Exo1" , "Tipin" , "Dscc1" , "Blm"  ,"Casp8ap2","Usp1" , "Clspn" , "Pola1" , "Chaf1b" , "Brip1" ,"E2f8")

DotPlot(SCIV,features= sgenes, split.by = "group") + theme(axis.text.x = element_text(angle = 90, vjust = 0.5, hjust=1))


g2m.genes <-c("Hmgb2","Cdk1","Nusap1","Ube2c","Birc5","Tpx2","Top2a","Ndc80","Cks2","Nuf2","Cks1b","Tmpo","Cenpf" ,"Tacc3","Fam64a","Smc4","Ccnb2","Ckap2l","Ckap2","Aurkb","Bub1","Kif11","Anp32e","Tubb4b","Gtse1","Kif20b","Hjurp","Cdca3","Hn1","Cdc20","Ttk","Cdc25c","Kif2c","Rangap1","Ncapd2","Dlgap5","Cdca2","Cdca8","Ect2","Kif23","Hmmr","Aurka","Psrc1","Anln","Lbr","Ckap5","Cenpe","Ctcf","Nek2","G2e3","Gas2l3","Cbx5","Cenpa")

DotPlot(SCIV,features= g2m.genes, split.by = "group") + theme(axis.text.x = element_text(angle = 90, vjust = 0.5, hjust=1))
